# Development, validation, and testing of an agriculture support exoskeleton in adjunct to conventional physiotherapy for farm labourers with chronic low back pain: A two-phase, mixed-methods, quasi-experimental pilot study protocol

**DOI:** 10.1371/journal.pone.0361352

**Published:** 2026-11-06

**Authors:** Gurjeet Kaur, Irshad Qureshi, Ashutosh Bagde, Bhupesh Sarode, Dhananjay A. Jolhe, Vinod M. Nistane

**Affiliations:** 1 Department of Musculoskeletal Physiotherapy, Ravi Nair Physiotherapy College, Datta Meghe Institute of Higher Education and Research (DU), Wardha, Maharashtra, India; 2 Department of Neuro Physiotherapy, Ravi Nair Physiotherapy College, Datta Meghe Institute of Higher Education and Research (DU), Wardha, Maharashtra, India; 3 Department of Biomedical Engineering, Faculty of Engineering and Technology, Datta Meghe Institute of Higher Education and Research (DU), Wardha, Maharashtra, India; 4 Bio Innovation Lab, Jawaharlal Nehru Medical College, Datta Meghe Institute of Higher Education and Research (DU), Wardha, Maharashtra, India; 5 Department of Mechanical Engineering, Visvesvaraya National Institute of Technology, Nagpur, Maharashtra, India; Nitte (Deemed to be University), Nitte Institute of Physiotherapy (NIPT), INDIA

## Abstract

**Background:**

Low back pain (LBP) affects 619 million people globally, with projections reaching 843 million by 2050. Farm labourers exhibit markedly higher prevalence than the general population, with lifetime (70–85%), 12-month (15–45%), and point (approximately 30%) prevalences driven by sustained trunk flexion, repetitive manual handling, and limited access to ergonomic or rehabilitative interventions. While conventional physiotherapy is the cornerstone of chronic LBP (CLBP) management, its effectiveness may be limited by ongoing mechanical loading during agricultural work. Back support exoskeletons (BSEs) have shown promise in reducing lumbar loading. However, no study has integrated exoskeleton development, validation, and clinical testing within a physiotherapy framework for farm labourers. The objective of the two-phase protocol is to design, develop, and laboratory-validate an agriculture support exoskeleton (AgSuEx), followed by examining the effects of AgSuEx-assisted physiotherapy compared with conventional physiotherapy alone on pain intensity, work productivity, disability, physical function, and quality of life (QOL) among farm labourers with CLBP.

**Methods:**

The two-phase, mixed-methods, quasi-experimental pilot study will be conducted in Wardha district, Maharashtra, India. Phase I will involve participatory co-design, iterative engineering development, and laboratory validation of an AgSuEx using finite element analysis (FEA), multibody dynamics (MBD) simulation, surface electromyography (sEMG), inertial measurement units (IMUs), interface pressure mapping, and validated usability instruments, integrated with focus group discussions (FGDs) from farm labourers. Phase II will employ a quasi-experimental, non-equivalent control group pretest-posttest design with repeated measures. Farm labourers aged 25–49 years with CLBP will be assigned (1:1) to AgSuEx-assisted conventional physiotherapy or conventional physiotherapy alone for 6 weeks, with follow-up at 3 and 6 months.

**Outcomes:**

Phase I primary outcome: lumbar extensor sEMG activation. Phase II primary outcomes: pain intensity (Quadruple VAS) and work productivity. Secondary outcomes: functional disability (RODI), physical function (RMDQ), and QOL (SF-12). Device-specific outcomes (SUS, QUEAD and LPP) will be assessed across both phases. Outcome assessors and data analysts will be blinded to group assignment.

**Conclusion:**

This protocol will generate preliminary evidence on the feasibility, usability, and preliminary clinical effects of integrating a Make in India AgSuEx with conventional physiotherapy for farm laborers with CLBP. Findings will guide future larger-scale controlled trials and inform ergonomic and rehabilitative strategies for agrarian populations.

**Trial registered prospectively:**

Clinical Trial Registry- India CTRI/2025/11/097725 [Registered on: 20/11/2025]. Acknowledgement Number: REF/2025/11/116856. URL: https://ctri.nic.in/Clinicaltrials/pmaindet2.php?EncHid=MTQ1NzE3&Enc=&userName=Protocolversion4.0,4/09/2026.

## Introduction

Agriculture remains one of the most physically demanding occupations globally, engaging nearly two billion people across Asia [1]. India, as an agriculture-based country with the largest agricultural workforce globally (65% of the Indian population), constitutes the backbone of the national economy, which contributes about 18.2% to the national GDP with an average annual growth rate of 4.18% over the past five years [1], where farming remains labour-intensive and dependent on human effort [2]. Maharashtra is a nationally prominent agricultural state, ranking fifth in agricultural output and first in cotton productivity [3,4]. Despite its agricultural prowess and advances in field mechanisation, agriculture has not become a pain-free occupation. Men and women alike experience prolonged trunk flexion, repetitive manual handling, and prolonged upright bending, regardless of the harvest season [5].

Manual harvesting and intensive manual picking have long been among the most common and uncomfortable agricultural tasks, and crops such as wheat and rice are mainly grown in India [5,6]. In-field work, which involves prolonged upright bending, causes static and repetitive loads to accumulate on the lumbar spine, leading to muscle elongation, ligamentous strain, mechanical deformation, mechanical degeneration, and resulting tissue damage [7–10]. Over time, these biomechanical stresses progress to chronic musculoskeletal conditions, including chronic Low Back Pain (CLBP), degenerative disc disease, spinal instability, and, in severe cases, surgical intervention [11–15]. Work-related musculoskeletal disorders (WMSDs) involving the spine and lower extremities rank alongside malaria, child mortality, and maternal health issues in rural health burdens, which are highly prevalent among farm labourers [16], producing both short-term consequences (impaired performance and daily activities) and long-term effects (permanent disability, job loss, and severe socio-economic consequences) [17,18].

LBP is defined by the WHO as pain between the lower twelfth ribs and the gluteal folds, with or without radiating pain. According to the NIH Research Task Force, CLBP persists for at least 3 months on half the days over 6 months [19]. Globally, LBP is the 4^th^ leading cause of disability, affecting 619 million people aged 25–49 [20,21] 37% of manual labourers [22], and with a lifetime 70–85%, 12-month 15%−45%, and a point prevalence of approximately 30%, making it a serious public health concern [23]. Farm labourers exhibit a higher prevalence than the general population [22,24]. In India, musculoskeletal disorders (MSDs) are the 2^nd^ & 4^th^ most common causes of outpatient visits and out-of-pocket expenditure among non-communicable diseases (NCDs) [25]. At the same time, Maharashtra reports the second-highest DALYs per 100,000 population attributable to LBP nationally [26]. Despite this burden, farm labourers continue traditional work methods with limited ergonomic education and access to modern technologies; their occupational health remains largely under-addressed [27], leading to the development of WMSDs [28]. Despite its high prevalence, the healthcare infrastructure for rural patients remains poor. Farm workers encounter several barriers to the right and timely treatment, including low wages, remoteness, limited access to physiotherapy services, and the use of traditional healing practices [8,29–31]. Early-stage LBP is commonly managed at home using massage, ointments, or traditional remedies, while more severe cases often prompt visits to private practitioners for analgesics and injections [32,33]. Public health centres in rural Maharashtra provide only symptomatic pain relief [29] and overdependence on medications / invasive treatments. Delayed treatment contributes to prolonged suffering, reduced productivity, and work absenteeism ranging from 1–10 days to several months, leading to significant income loss and high out-of-pocket healthcare expenditure. Annual costs related to back and extremity pain have been reported to exceed ₹9 lakhs in rural populations, despite daily wages averaging approximately ₹100 [29,32–34].

Growing research findings on the management of LBP demonstrate the benefits of pharmacological and non-pharmacological approaches (physiotherapy). However, conventional physiotherapy encompassing back care education, behavioural treatment, exercise therapy, electrotherapy, manual therapy, braces, yoga, acupuncture, and traction is considered the cornerstone of CLBP rehabilitation [35,36], for pain relief, core stabilisation, spinal mobility, and functional restoration [37,38]. However, these interventions primarily address impairments outside the work environment and do not adequately mitigate continuous mechanical loading during agricultural work. Without modifying the physical demands of farming tasks, long-term recurrence and progression of symptoms remain common [36]. In recent years, wearable assistive technologies (Back Support Exoskeletons- BSEs) have emerged as promising ergonomic solutions to reduce lumbar muscle activation, fatigue, and perceived exertion during physically demanding tasks [7]. BSEs were initially developed for military, rehabilitation, industrial, or clinical applications in high-income countries. Despite their growing applications, their adaptation to agricultural contexts remains underexplored [39]. However, commercially available systems (Ekso Bionics, Laevo, Otto Bock, etc) are complex, bulky, sensor-intensive, impose high-power demands, require extensive maintenance, offer limited adaptability, and are prohibitively expensive for rural agricultural settings in low- and middle-income countries [40–45]. Moreover, prolonged BSE use without concurrent active rehabilitation may risk trunk muscle deconditioning [46]. Most existing exoskeleton research has been laboratory-based, short-term, and conducted without the involvement of end users (farm labourers), limiting its real-world applicability and adoption [47]. Therefore, the literature suggests that adapting and refining existing platforms offers a feasible pathway to create affordable, agriculture-specific exosuits that better meet the needs of rural workers [7,40].

The growing body of research identified two critical gaps that underpin this protocol. First, a “*practical knowledge gap*” exists, as available research lacks participatory ergonomics, biomechanical interventions, collaboration between developers/end-users/stakeholders, and clinical testing of an affordable, agriculture-specific BSE within a physiotherapy framework for Indian farm labourers tailored to real-world farming tasks. Most are lab-based and lack real-world testing. Second, a “*population gap*”: farm labourers remain underrepresented in exoskeleton research, and interventions have not been designed to address the unique ergonomic profile of prolonged upright bending in farming tasks. To the best of our knowledge, the combined effectiveness of physiotherapy with an agriculture support exoskeleton (AgSuEx), which offers potential solutions, remains unexplored. These limitations highlight the need for tailored, community-based interventions for postural correction, rehabilitation, and periodic ergonomic training, as well as for expanding low-cost, farmer-sensitive research to the broader rural context of Maharashtra. This study will address these gaps by adopting a participatory, “by the farmers, for the farmers” approach, involving farm labourers in the co-development and evaluation of a farmer-friendly AgSuEx to redistribute lumbar load during prolonged upright bending, ensuring flexibility, comfort, and usability across various tasks, such as harvesting in Wardha district, Maharashtra. By combining rehabilitation with task-specific mechanical support, this protocol would generate an evidence-based, scalable solution for LBP prevention, protection and slowing down further progression [44], aligning with the “Make in India Initiative” to ensure acceptance in low-resource rural settings [7,9,48], and to reduce dependency on medical treatment and promote pain-free farming for a sustainable, context-appropriate intervention that may reduce pain, disability, and work-related strain while promoting long-term occupational health and productivity among farm labourers.

### Aims, objectives, and hypotheses

#### Phase I: Design, Development, and Laboratory Testing of AgSuEx in a Simulated Environment.

**Hypothesis:** An AgSuEx will achieve a System Usability Scale (SUS) score of ≥68 (the established threshold for acceptable usability) and report acceptable comfort ratings during lab-based testing; lumbar biomechanical load with the AgSuEx will be significantly lower than without it among farm labourers with CLBP during prolonged upright bending tasks.

**Aim:** To design, develop, and evaluate an AgSuEx for farm labourers with CLBP to reduce lumbar spinal load during harvesting, through controlled laboratory testing in a simulated environment.

**Objectives:** (1) To evaluate mechanical integrity, stress distribution, deformation and postural compatibility of the AgSuEx using multibody dynamics (MBD) and finite element analysis (FEA); (2) To conduct laboratory-based testing and re-testing, including static load testing, structural validation, muscle activation patterns, trunk kinematics, interface pressure distribution, postural balance, and user-reported usability and comfort during simulated upright bending tasks with and without the AgSuEx to ensure safety and biomechanical compatibility.

#### Phase II: Pilot Testing and evaluation of an AgSuEx Integrated with Conventional Physiotherapy Vs Conventional Physiotherapy alone in Farm labourers with CLBP.

**Phase II exploratory expectation:** As Phase II is an exploratory pilot study not powered for confirmatory hypothesis testing, no formal null and alternative hypotheses are specified. Instead, Phase II is guided by a directional expectation: that AgSuEx-assisted conventional physiotherapy will show a pattern of greater improvement in pain intensity, work productivity, functional disability, physical function, and quality of life than conventional physiotherapy alone among farm labourers with CLBP during the harvesting period. This expectation will be evaluated through estimated between-group differences and their 95% confidence intervals, as detailed under Statistical Analysis, rather than through significance testing of a null hypothesis.

**Aim:** To evaluate and compare the preliminary effects of an AgSuEx-assisted conventional physiotherapy versus conventional physiotherapy alone on pain, work productivity, disability, physical function, and QOL among farm labourers with CLBP.

**Objectives:** (1) To determine the preliminary effects of an AgSuEx combined with conventional physiotherapy on all primary and secondary outcomes; (2) To determine the preliminary effects of conventional physiotherapy alone on all primary and secondary outcomes; (3) To compare the preliminary effects between groups on all primary and secondary outcomes across all time points among farm labourers with chronic LBP.

As a pilot study, Phase II is not powered to test definitive efficacy hypotheses. The reported estimates are preliminary and intended to inform the design and sample size of a future, adequately powered, definitive randomised controlled trial. No confirmatory efficacy conclusions will be drawn from Phase II findings.

## Methods

### Trial design and reporting framework

This study will be conducted as a two-phase, mixed-method, quasi-experimental pilot study. Phase I will include the participatory co-design, iterative engineering development, and laboratory validation of the AgSuEx, using simultaneous qualitative data collection from FGDs with farm labourers, as presented in accordance with Mixed Method Reporting in Rehabilitation & Health Sciences (MMR-RHS) [45] (S1 Appendix) and the Consolidated Criteria for Reporting Qualitative Research (COREQ) checklist [49] (S2 Appendix), with a focus on usability, comfort, safety, and biomechanical feasibility as measured by simulating in a laboratory or within the campus. Phase II will employ a quasi-experimental, non-equivalent control group pretest-posttest design with repeated measures, reported in accordance with the Transparent Reporting of Evaluations with Non-Randomized Designs (TREND) checklist [50] (S3 Appendix), the Standard Protocol Items: Recommendations for Interventional Trials (SPIRIT) [51] (S4 Appendix), the Template for Intervention Description and Replication (TIDieR) [52] (S5 Appendix), and the complete, structured focus group interview guide is elaborated in Appendix S6. The quasi-experimental design was selected because random allocation is not feasible in agricultural field settings during harvesting seasons, while still enabling prospective comparison of intervention effects over time under real-world conditions.

This study would employ a sequential exploratory mixed-methods design, denoted QUAL → QUAN → qual, in which the qualitative strand is initiated first to explore, discover, and generate insights that directly shape the subsequent quantitative phases. In the sequential exploratory design used here, qualitative data will be collected and analysed at the outset, with the findings used to inform, refine, or co-develop the quantitative instrument, tool, or intervention, which will then be tested. In this protocol, pre-field Focus Group Discussions (FGDs) with farm labourers will be conducted first (QUAL strand) to explore gender-specific postural demands, lived ergonomic experiences, healthcare-seeking behaviours, and feasibility barriers, insights that directly shape the AgSuEx design specifications, conceptualisation, and co-design priorities. The dominant quantitative strand (QUAN phase) then follows, encompassing Phase I biomechanical laboratory validation of the co-designed AgSuEx and Phase II quasi-experimental clinical testing. Post-field FGDs (qualitative strand) will be conducted after field evaluation and clinical testing to contextualise, elaborate on, and enrich the interpretation of quantitative findings with real-world user experiences and implementation insights. The qualitative strand is prioritised and positioned first; the quantitative strand is dominant in volume, analytical weight, and hypothesis-testing function. Integration of the two strands will occur at three pre-specified points: (1) at the development interface, where pre-field FGD findings directly inform Phase I AgSuEx design specifications and co-design priorities; (2) at the analysis stage, where a joint display integration matrix will map quantitative biomechanical and clinical findings against qualitative user-experience themes, per MMR-RHS guidelines; and (3) at interpretation, where post-field FGD qualitative findings contextualise, elaborate, and explain the quantitative Phase II clinical outcomes.

The planned integration procedure at each point is described as follows. At Point 1 (development interface), key themes emerging from pre-field FGD thematic analysis, including postural demands, comfort priorities, cultural acceptability, and anticipated barriers to device use, will be converted into structured design criteria and presented to the engineering team (VNIT) in a formal co-design workshop before prototype fabrication begins. Any resulting design modifications will be documented in a change log to create an auditable link between qualitative findings and engineering decisions. At Point 2 (analysis integration), a joint display matrix will be constructed in tabular form, with each row representing a domain of interest (e.g., lumbar load reduction, usability, fatigue), and the columns presenting the corresponding quantitative outcome (sEMG or clinical scale result) alongside the thematic finding from the matched FGD question. Convergence, complementarity, or divergence between the two data types will be identified explicitly, and discordant findings will be discussed and explained rather than suppressed. At Point 3 (interpretation), post-field FGD themes will be applied to each primary and secondary quantitative finding to provide contextual explanation: for example, if pain scores improve but work productivity does not, FGD data will be examined for participant-reported barriers to continued farming productivity despite reduced pain. The final integrated interpretation will be presented in the Results section of the study report using a narrative synthesis approach structured around the three integration points, supplemented by illustrative participant quotes and a summary joint display table. This three-point integration structure operationalises the MMR-RHS framework and ensures that the qualitative and quantitative strands function as genuinely integrated components rather than parallel, unconnected analyses.

### Study setting and population

#### Trial status.

This is protocol version 4.0, 4/09/2026. The study has not yet commenced, and no participants have been enrolled at the time of manuscript submission (CTRI recruitment status: not yet recruiting). (A) *Participant recruitment:* Recruitment is scheduled to begin in November 2026. Phase I healthy volunteer recruitment (n = 12) is planned for November 2026. Phase II farm labourer recruitment (n = 24) is planned for February 2027. (B) *Data collection:* Phase I biomechanical laboratory testing and associated focus group discussions (FGD) are anticipated to be completed by January 2027. Phase II intervention delivery, clinical outcomes assessments, and post-field FGD are estimated to be completed by November 2027, following the final 6-month follow-up assessment of the last enrolled participant. (C) *Results expected:* Phase I results are expected to be available for dissemination by early 2027. Final results from Phase II, including primary and secondary clinical outcomes across all time points, are expected by December 2027.

The Study will be conducted in rural agricultural communities of Wardha district, Maharashtra, India, where nearly 79% of its workforce is engaged in agriculture (Census 2011) and is economically vulnerable, with a gross district domestic product (GDDP) below the state average [53]. This exemplifies challenges in socio-economic and health inequities, as the Government of India, under NITI Aayog, identified 101 aspirational districts, including four districts in Maharashtra [54]. Conventional physiotherapy sessions and baseline assessments will be conducted at accessible community locations or at affiliated physiotherapy facilities linked to the Community Health Care Program (CHCP) of Ravi Nair Physiotherapy College (RNPC), Datta Meghe Institute of Higher Education and Research (DMIHER) (DU), serving the villages of Wardha district. Laboratory-based design, testing, and biomechanical validation activities for the AgSuEx will be conducted in collaboration with the engineering facilities at the Department of Mechanical Engineering, Visvesvaraya National Institute of Technology (VNIT), Nagpur. This combination of community-based and institutional settings is designed to ensure both the ecological validity of the clinical intervention and the rigorous technical evaluation of the device.

Under the Agricultural Mechanisation Scheme (2021–22), Wardha ranked 19th out of 34 districts in mechanisation adoption, with benefits largely limited to basic equipment such as tractors, seed drills, threshers, rotavators, and plows [55]. High-level ergonomic or assistive technologies are still lacking. The Agricultural Technology Management Agency (ATMA) in Wardha district sought to increase awareness of modern farming methods, ergonomic principles, and various farming methods. Yet farm labourers were less engaged, often viewing farming as a job that did not require formal training. This resistance persists despite significant economic lag, low mechanisation penetration, and ongoing health burdens [56,57]. The target population for Phase I will be healthy volunteers, and that of Phase II will comprise male and female farm labourers aged 25–49 years with CLBP who are actively engaged in harvesting tasks. In Phase II, eligible farm labourers will be allocated to one of two parallel groups at a 1:1 allocation ratio: (1) conventional physiotherapy combined with the AgSuEx, or (2) conventional physiotherapy alone.

### Eligibility criteria

**Inclusion criteria:** Phase I healthy farm labourers actively engaged in agricultural work (no CLBP). Phase II adult farm labourers aged 25–49 years with a diagnosis of CLBP persisting ≥3 months and present on at least half of the days in the past 6 months, as per the WHO. According to the NIH Research Task Force, CLBP is localised between the twelfth thoracic vertebra (T12) and the gluteal fold [58]. Eligible participants must report moderate pain intensity, defined as a Visual Analogue Scale (VAS) score of 3–7 at baseline [59]. Participants must be actively engaged in agricultural work for ≥7 hours per day, ≤ 5 days per week and have at least ≥5 years of farming experience, and be working as farm labourers during the harvesting period (February-March) [44]. All participants must be willing to participate and provide written informed consent in Marathi, Hindi, or English. Participants will also be required to demonstrate clinical features consistent with mechanical or movement-related LBP with one or more of the following characteristics: LBP that worsens with sustained end-range movements or postures; evidence of lumbar hypermobility on segmental motion assessment; mobility deficits of the thoracic spine or lumbo-pelvic/hip regions; reduced trunk or pelvic-region muscle strength or endurance; or movement coordination impairments during performance of community, work-related, recreational, or occupational activities. Sacroiliac joint dysfunction will be considered present when at least three of five standard provocative manoeuvres are positive, with either the thigh thrust test or the compression test mandatory among the positive findings [60].

**Exclusion criteria:** Participants will be excluded if they have had lumbar spine surgery within the previous 12 months or are scheduled for surgery within the next 12 months. Presence of radiating LBP, positive straight leg raise or slump tests indicating neural tension, fractures, neurological disorders, or participation in structured spine-specific exercise training within the three months preceding study enrolment. Individuals with uncontrolled mental health conditions that may impede participation or contraindicate physical activity will also be excluded. Further exclusions include inflammatory arthritis, malignancy, pregnancy, or childbirth within the past 6 months, severe musculoskeletal deformities or any condition contraindicating the safe use of an exoskeleton device. Agricultural workers employed primarily as office staff, truck drivers, recent migrants, landlords not actively involved in field work, or individuals not engaged in agricultural work during the preceding year will not be eligible. Participants currently using medications that may influence neuromuscular or physiological responses, such as anticoagulants or systemic corticosteroids, will also be excluded.

**Eligibility criteria for personnel delivering the intervention:** The interventions will be delivered by a multidisciplinary research team to ensure clinical safety, protocol fidelity, and technical accuracy. Licensed physiotherapists will provide conventional physiotherapy with a minimum bachelor’s degree in physiotherapy and at least 2 years of clinical experience in a musculoskeletal rehabilitation program. The physiotherapists will receive training in the study-specific intervention protocol, including standardised exercise prescription, progression criteria, and documentation processes, before participant recruitment. A fit, training, and supervision of the AgSuEx will be conducted by the trained research assistants (final-year physiotherapy students) under the direct supervision of a licensed physiotherapist and in consultation with VNIT biomedical and mechanical engineers. All research assistants will be trained on procedures for fitting an exoskeleton, conducting safety checks, instructing participants, monitoring for adverse events, and using standard operating procedures. Engineering personnel will provide technical oversight of device integrity, maintenance, and safety, but will not be involved in clinical outcome assessment or in the allocation of interventions.

### Sample size

As this is an exploratory pilot study, formal power-based sample size calculation is not the primary objective [61]. A sample of 12 participants per group (for feasibility and budgetary constraints), n = 24 total in Phase II, will be employed, consistent with the widely cited rule of thumb for pilot studies (n = 12 per group) [61–63] and with pilot study guidelines recommending 10–30 participants per arm [63]. For Phase I laboratory testing, simulated farming environment, and real field testing, 12 healthy farm labourers will participate. This sample size was selected to estimate feasibility parameters (recruitment, retention, adherence, and acceptability) and to generate preliminary effect-size estimates, means, standard deviations, and attrition rates with associated confidence intervals, which will together be used to calculate the sample size required for a future, adequately powered, definitive randomised controlled trial.

### Recruitment and informed consent

Participants will be recruited from Wardha district using convenience sampling through collaboration with local community leaders, agricultural groups, and healthcare outreach networks. Recruitment will be timed to coincide with the wheat harvesting season to maximise feasibility and ecological validity. Initial screening will be conducted by trained research personnel through brief interviews and clinical assessment. Reasons for non-participation, ineligibility, or refusal will be recorded to inform external validity.

Written informed consent will be obtained by the principal investigator (PI) or a trained team member fluent in English, Hindi, and Marathi before enrolment. Consent forms will be made available in each of the three languages. Verbal and Written information about the purposes of the study, procedures, risks and benefits, voluntary participation, and the right to withdraw at any point free from financial costs will be provided to the participants. All participants will have sufficient time to ask questions before providing written consent.

### Ethical approval and considerations

The study protocol was approved by the Institutional Ethics Committee (IEC) of DMIHER (DU), Re-Accredited with NAAC Grade A++, Wardha, Maharashtra, India [DMIHER(DU)/IEC/2025/546, and prospectively registered with the Clinical Trial Registry- India CTRI/2025/11/097725 (Trial REF/2025/11/116856; Registered: 20 November 2025; URL: https://ctri.nic.in/Clinicaltrials/pmaindet2.php?EncHid=MTQ1NzE3&Enc=&userName=). All procedures will comply with the Declaration of Helsinki and institutional ethical guidelines. To protect confidentiality, each participant will be assigned a unique code comprising a prefix that identifies the control (C) or intervention group (I) and a consecutive number. Codes, and thus identities, will be stored securely in a password-protected file, with access limited to the authorised researcher responsible for coding, who will not be involved in subsequent qualitative analysis. Electronic data will be stored in password-protected systems; physical data will be stored in locked cabinets and accessible only to relevant study staff.

### Protocol amendments and dissemination

Any significant changes to the study protocol that affect scientific integrity, participant safety, eligibility criteria, intervention procedures, outcome measures, or data analysis will be properly documented, reviewed by the PI and co-investigators, and submitted to the IEC for approval before initiation. Amendments will be communicated to collaborating institutions and uploaded to the trial registry.

Findings will be disseminated through peer-reviewed publication and presented at national and international conferences on physical therapy, occupational health, and agricultural ergonomics. Results will also be disseminated to the communities actively involved in the study in terms they can understand. As per the institution’s data-sharing policy, data will be released upon a reasonable request.

### Participant timeline

**Phase I:** Each participant will complete all assessments during a single study visit, comprising eligibility screening, written informed consent, baseline demographic data collection, a 10-minute familiarisation period with the AgSuEx, and standardised upright bending tasks under two conditions (without and with the AgSuEx). A 10-minute rest period will separate task conditions. Usability and comfort questionnaires will be completed after the task.

**Phase II:** Following eligibility screening and informed consent, participants will undergo baseline assessment before commencing the six-week intervention. All assessments and intervention procedures will be conducted according to a predefined schedule. No washout periods are planned, reflecting real-world clinical practice. The SPIRIT-compliant schedule of enrolment, interventions, and assessments is presented as Fig 1.

**Fig 1:**
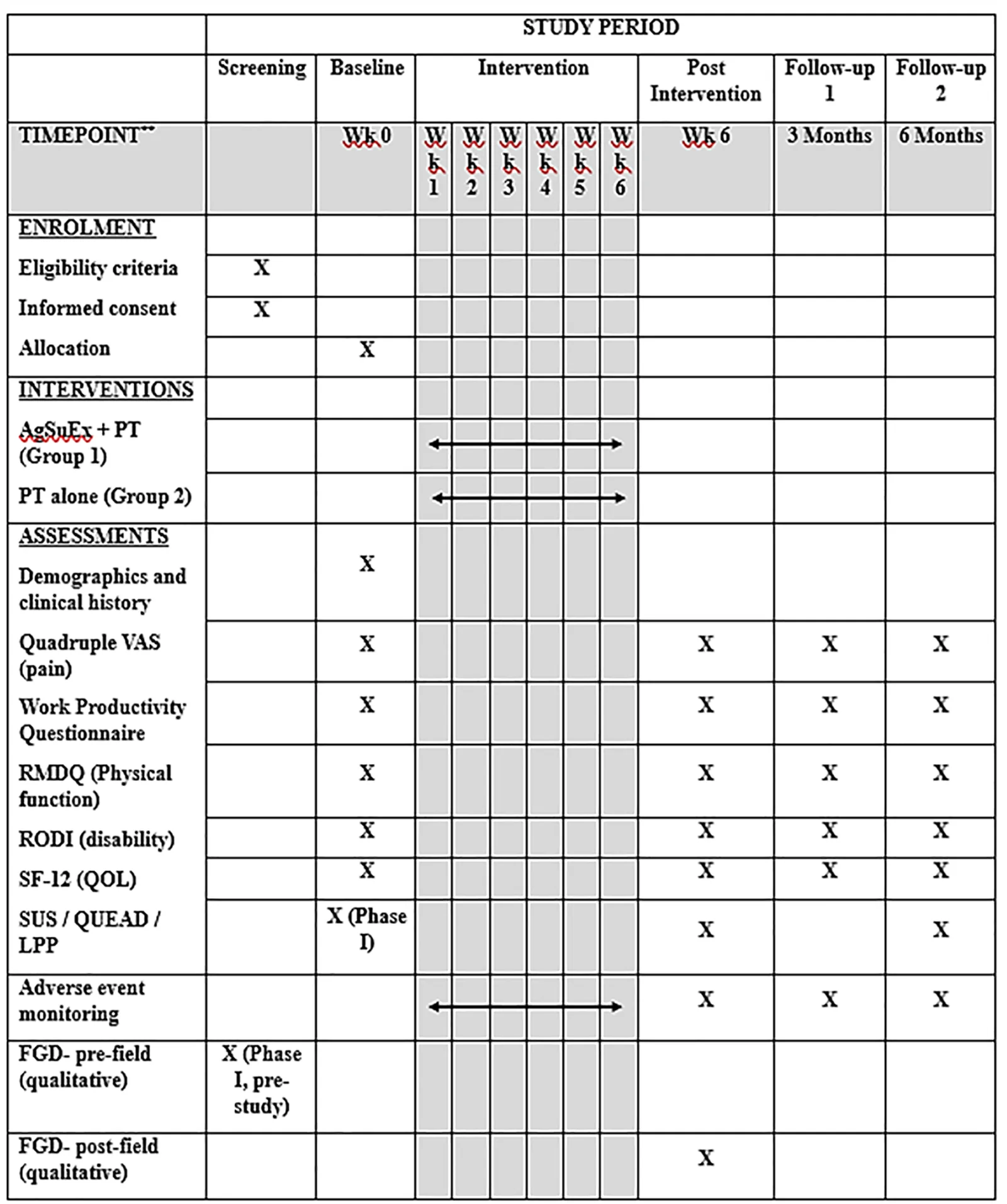
SPIRIT schedule of enrolment, interventions, and assessments for Phase II (participant timeline).

### Study procedure

The proposed 2-phase study model (Fig 2) meets the FINER criteria for a good research design. “*Feasibility*” is supported by a phased approach in which Phase I will involve VNIT, Department of Mechanical Engineering, Nagpur, RNPC, Faculty of Engineering and Technology (FEAT), DMIHER, Wardha, as the collaborators of this research are on the design, prototyping, and testing of the AgSuEx. Phase II will involve implementation among farm labourers with CLBP recruited through rural health networks and community outreach to ensure accessibility and adherence. The research is clinically “*Interesting*” and methodologically new, linking engineering, physiotherapy, and participatory research to the field. Involvement of farm labourers in the planning also fosters engagement among stakeholders and greater applicability to real-world scenarios, alongside an interdisciplinary team of researchers, engineers, physiotherapists, and mentors. The project is “*Novel*” in its integrative approach. It is the product of a novel amalgamation of physiotherapy concepts and a farmer-led AgSuEx, producing a context-specific approach to CLBP that addresses a major gap in both rehabilitation and occupational health research. “*Ethical*” feasibility is maintained by following established research protocols, informed consent protocols, risk minimisation, benefit maximisation, and organised safety and adverse events tracking. The study is highly “*Relevant*” to clinical and public health practice, as it offers a scalable, non-invasive strategy for addressing WMSDs, QOL, work productivity among farm labourers, and agricultural ergonomics.

**Fig 2:**
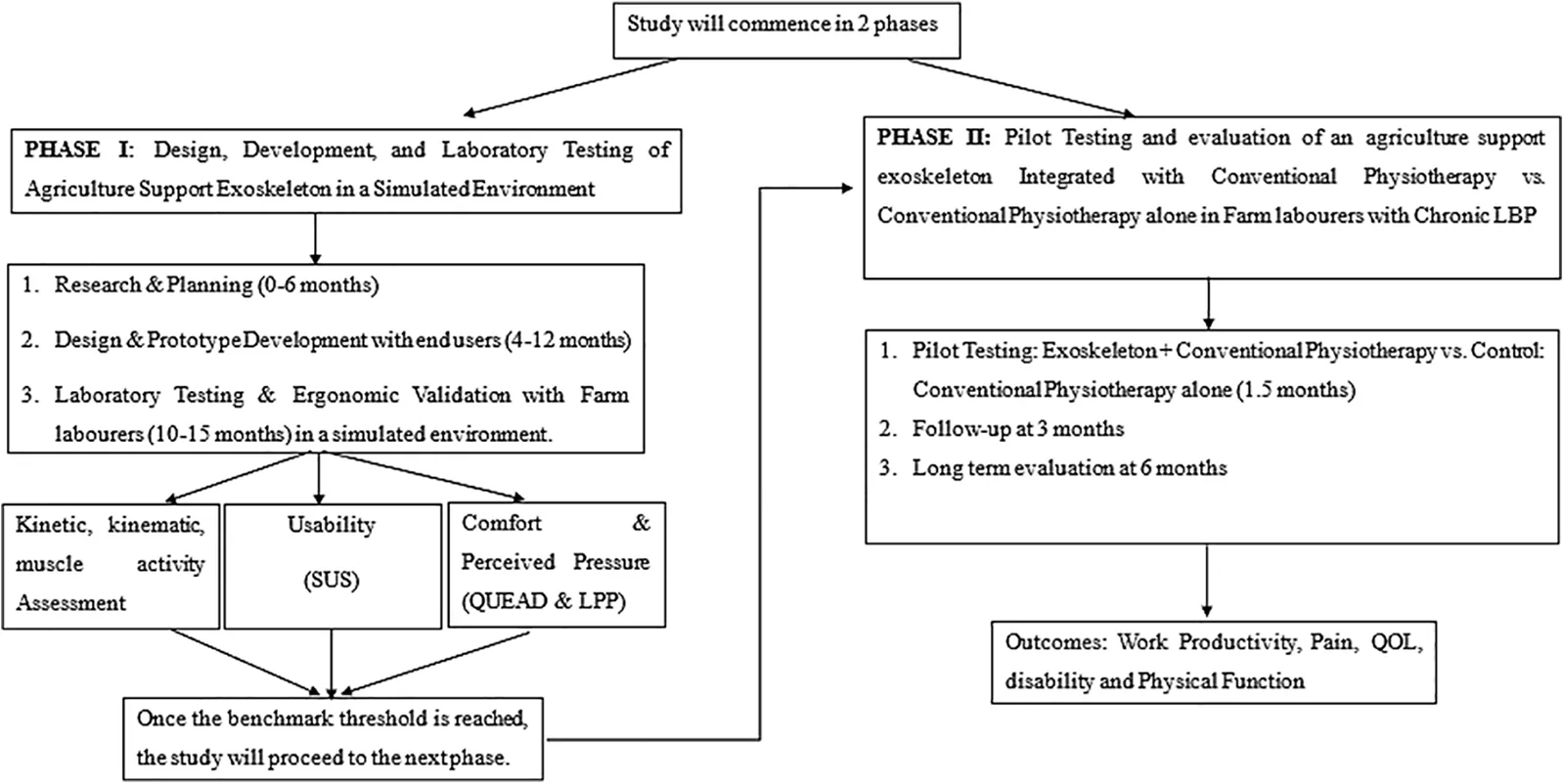
Flow chart of the two-phase study.

#### Phase I: Design, Development, and Laboratory Testing of Agriculture Support Exoskeleton in a Simulated Environment.

**Methodological orientation:** A phenomenological approach will be adopted for the qualitative strand to explore the lived experiences of farm labourers regarding occupational biomechanical demands, health-seeking behaviour, and perceived ergonomic needs. This approach was selected because it enables in-depth exploration of participants’ subjective experiences and perceptions that cannot be captured solely through instruments.

**Research team and reflexivity:** FGDs will be facilitated by the PI (GK), a Junior Research Fellow in Musculoskeletal Physiotherapy with postgraduate training in qualitative methods and no prior clinical relationship with the participants. A second researcher (a trained research assistant and a final-year physiotherapy student) will serve as the note-taker and recorder. Both researchers are female. Participants will be informed of the researcher’s academic affiliation and study objectives before consent. A reflexivity journal will be maintained throughout data collection and analysis to document and critically examine the researcher’s assumptions, positionality, and potential influence on data interpretation.

**FGD design:** Pre-field FGDs will be conducted before device development to explore the gender-specific postural and physical demands, healthcare access, perceived ergonomic needs, and barriers to rehabilitation. Post-field FGDs will be conducted following the field evaluation of the AgSuEx to assess user experiences regarding comfort, perceived support, usability, workload reduction, social acceptability, and recommendations for device improvement. Two FGDs will be conducted (one male and one female group, each with 8–9 participants). Sessions will last 1 hour and be facilitated by a semi-structured question guide developed by the research team and validated. No non-participant observers will be present. Sessions will be audio-recorded with signed consent, and field notes will be taken concurrently.

**Data analysis (planned):** FGD recordings will be transcribed verbatim in Marathi, translated into English, and analysed using the inductive thematic analysis framework of Braun and Clarke (2006) [64] by two independent coders (GK and a co-investigator). A coding tree will be developed inductively from the data. Disparities will be resolved by consensus or referral to a third investigator. Coders and themes will be managed in R software version 3.4.2. To check for member checking, we will provide a summary of major themes to a subsample of interviewees to confirm accuracy and coherence. We will assess data saturation by measuring repetition of themes across FGD transcripts.

### Patient and public engagement

Farm labourers and community stakeholders will participate in and understand the design and design assessment of the AgSuEx through pre-field and post-field FGDs. Feedback on common work postures, task-related demands, comfort preferences, and perceived barriers to device use will be systematically used to develop and refine the devices. The participatory nature of these methods ensures that the AgSuEx design and intervention protocols align with agricultural practices and user expectations. Community interactions among community representatives and stakeholders will convey study aims and methods to the general public. While farm labourers will not participate in data analyses or authorship decisions, results will be released to the relevant communities in an accessible form consistent with the principles of transparency, relevance, and ethical engagement.

### Engineering development steps

**Step 1- System design and conceptualisation:** A detailed user and task analysis will be conducted using video recordings of farm labourers performing wheat harvesting tasks to quantify biomechanical load on the lumbar spine during upright bending (Fig 3). The conceptual framework draws from established orthotic devices [Jewett brace (thoracolumbosacral orthosis), SWASH bilateral hip orthosis, scapular retractor braces]. The AgSuEx will feature chest pad to unload the spine during upright forward bending postures, effectively distributing stress and also modular shoulder, trunk, pelvic, hip, and thigh units integrated with cushioned contact surfaces and an adjustable multi-angle locking mechanism at the hip joint supporting controlled lumbar flexion from 0° to 180° in 10° increments to offload spinal stress during repetitive forward-bending activities every day in agricultural work [47]. The engineering and material specifications are shown in Tables 1–3. Anthropometric measurements will be recorded using standardised anthropometric techniques to inform customised design specifications for Indian farm labourers [67,68].

**Table 1:** Design Characteristics of the AgSuEx.

Characteristics	Agriculture Support Exoskeleton (AgSuEx)
**Control strategy**	Hip-level multi-angle adjustable stay lock for controlled lumbar spine flexion (0°-180° in 10° increments)
**Degrees of Freedom**	Spine: 3; Hip: 3
**Type of sensors**	Kinematic sensors (IMUs), bioelectric sensors (sEMG), pressure sensors
**Task performed**	Upright bending tasks for wheat harvesting
**Device type**	Passive (no motorised/electronic components)
**Total weight**	Approximately 1 Kg
**Size**	Customised per individual anthropometric measurements
**Estimated Cost**	Rs 3000 (indigenously designed)
**Make in India**	Yes, indigenously designed for Indian agricultural workers

**Table 2:** Engineering Specifications of the AgSuEx [66].

Engineering Specification	Detail
**Material of frame (bars)**	Aluminium 6061-T6
**Cross-section of upper side frame (bars)**	20 X 6 mm (solid)
**Diameter of the lower thigh frame (bars)**	20 X 6 mm (solid)
**Length of the upper side frame (bars)**	17 inches
**Length of the lower thigh frame (bars)**	8 inches
**Type of bars**	Solid
**Bars thickness**	2 mm
**Shoulder straps**	2-inch wide, 26-inch long with a cushion for about 25 cm in length
**Chest Pad Material**	HDPE (High-Density Polyethylene)
**Chest pad dimensions**	Length 9 in × Width 8 in × Thickness 4 mm
**Pelvic belt circumference**	81 to 132 cm, 20-gauge thickness
**Pelvic belt cushion**	2.5 cm thick bilateral lumbar region padding
**Thigh cuffs**	Length- 11.4 cm; Arc length- 29 cm; 4 mm sheet; 2 cm Ethaflex cushioning
**Multi-Angle Stay Lock**	0°-180° in 10° increments; button operated; auto locks on release; Diameter- 8 cm

**Table 3:** Structural components of the AgSuEx [47].

Exoskeleton Component	Constraint	Link	Approximate Weight
**Shoulder support**	Rigid	Chest pad – upper side bars (posteriorly)	10 gm
**Trunk support**	Rigid	Chest pad – upper side bars (anteriorly)	700 gm
**Pelvic support**	Rigid	Back – abdomen interface	200 gm
**Hip joint (multi angle stay lock)**	Revolute locking	Pelvic support – thigh support	25 gm
**Thigh support**	Rigid	Thigh cuffs – lower side bars – humanoid thigh	100 gm
**Total**			**1,035 g ≈ 1 kg**

**Fig 3:**
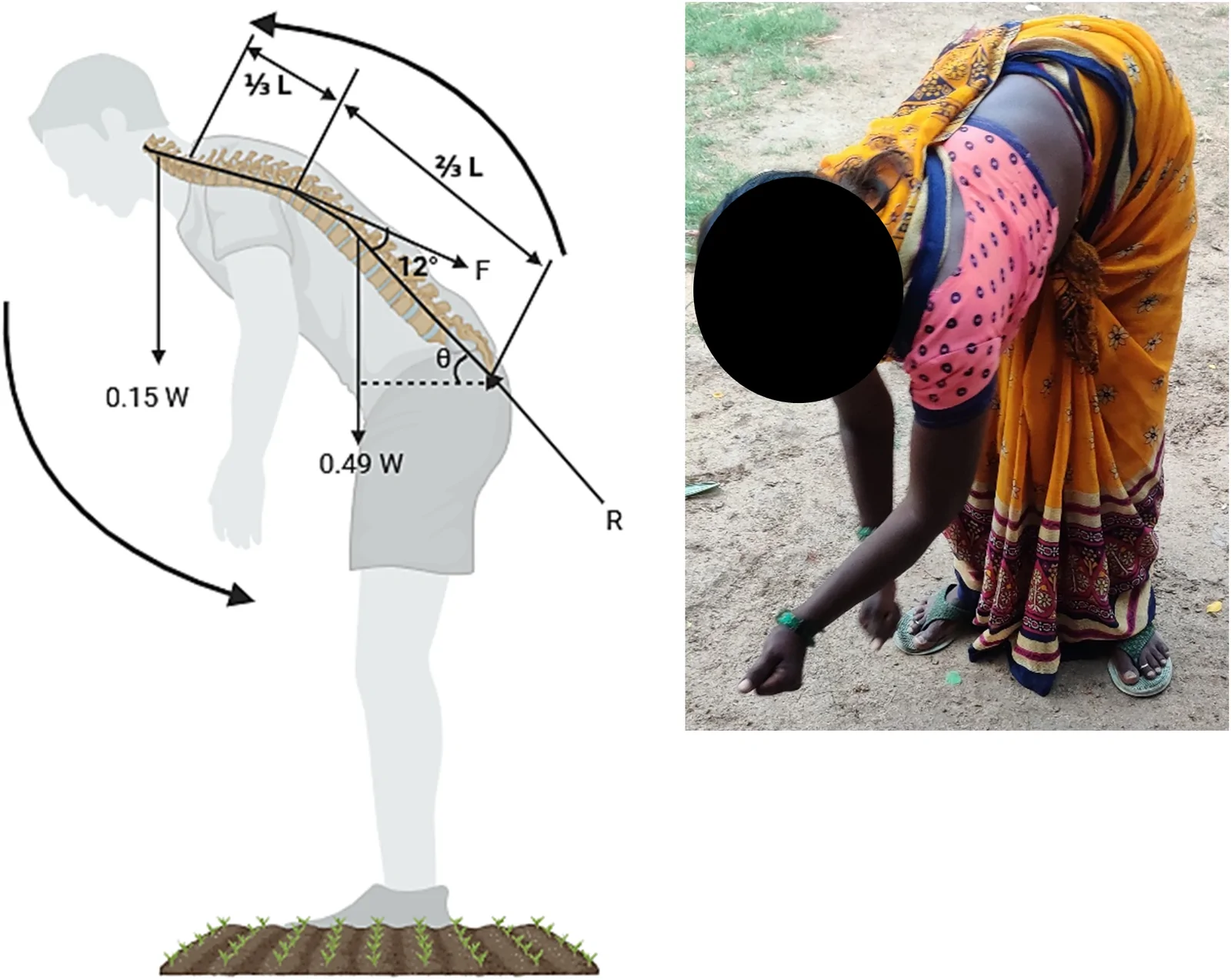
Upright bending posture and related load on the lumbar spine. This figure is similar to, but not identical to, the original image and is provided for illustrative purposes only; adapted from [65].

**Step 2- CAD modeling and virtual simulation:** A full-scale 3D virtual prototype will be developed in ANSYS 24 for dynamic and kinematic analysis during agricultural postures, assessing stress distribution, deformation, and potential failure points under physiological loading (VNIT, Nagpur) [69].

**Step 3- Realistic CAD and MBD:** The AgSuEx will be modified and upgraded to a more realistic CAD model, incorporating humanoid anthropometric segments. This model will then be exported to ANSYS 24, based on the humanoid dimensions as per Winter (2009), for motion analysis [47], to measure the positions and orientations of the humanoid and exoskeleton joints during simulation. These features will enable us to analyse the system’s behaviour while it is controlled and stable during the simulation process. Subsequently, lock-control strategies and real-time feedback on system dynamics under constant and disturbance forces will be explored. VNIT will take the lead in engineering simulations (FEA, MBD), structural safety analysis, and prototype technical validation.

**Step 4- Material selection and prototyping**: Structural components of the prototype will be fabricated from the following materials as described in Table 4. All the required material properties were verified and taken from the reference American Society for Metals (ASM) International, 2025 [70] and MATWEB datasheets to ensure the durability and reliability of the exoskeleton under field conditions (Al 6061-T6 [71], stainless steel [72], nylon [73], EVA [74], HDPE [75]). The prototype prioritises affordability, portability, self-repairability, and even contact pressure distribution. Modular fasteners, breathable harnesses, and adjustable strapping systems will provide comfort and field adaptability.

**Table 4:** Key modelling requirements and materials for fabrication.

SN.	Component	Material
1	Upper side frame bars (×2)	Al 6061-T6
2	Lower thigh frame bars (×2)	Al 6061-T6
3	Hip joint multi-angle stay lock (×2)	Stainless steel
4	Pelvic frame	Al 6061-T6
5	Chest pad	High-Density Polyethylene (HDPE) 4 mm with Ethylene Vinyl Acetate (EVA)
6	Thigh cuffs (×2)	4 mm HDPE sheet + 20 mm Ethylene Vinyl Acetate (EVA) inner layer
7	Cushioning – chest pad, thigh cuffs, lumbopelvic padding	Ethylene Vinyl Acetate (EVA)
8	Shoulder straps (×2)	Nylon webbing + Ethaflex cushion
9	Pelvic belt	20-gauge nylon webbing + foam padding
10	Shoulder straps	Nylon webbing + 250 mm EVA cushion

**Step 5- Laboratory testing and biomechanical validation:** Technical validation of both virtual and physical prototypes will be conducted at VNIT’s biomechanics laboratory. Twelve healthy farm labourers will perform standardised upright bending tasks simulating wheat harvesting, with and without the AgSuEx, separated by a 10-minute rest period to minimise fatigue effects [76]. Surface EMG [SENIAM (Surface EMG for Non-Invasive Assessment of Muscles) guidelines] [77] will assess back extensor muscles (erector spinae longissimus, erector spinae iliocostalis, and multifidus) using 10 mm electrodes at 20 mm inter-electrode distance. Maximal voluntary contraction (MVC) values will be obtained in a prone position through trunk extension. Following this, participants will perform forward bending tasks, holding the posture with knees straight (but not locked), with slight flexion permitted if necessary. The primary EMG parameters include root mean square (RMS) amplitude, mean power frequency (MPF), and median frequency (MF) for evaluating muscle activation and fatigue characteristics, as well as the extent of spinal unloading and the muscular relief provided by the device [39,78]. Specifications are described in Table 5. IMUs will be placed at T12, L1-L5, and S2 to track sagittal plane lumbar kinematics and postural transitions. Interface pressure distribution at exoskeleton-body contact regions will be assessed using pressure mapping sensors. User-reported outcomes will be collected using the System Usability Scale (SUS), Questionnaire for the Evaluation of Physical Assistive Devices (QUEAD), and Local Perceived Pressure (LPP) scale [48,79].

**Table 5:** EMG Electrode Placement Specifications and Parameters as per SENIAM Guidelines.

Muscle	Electrode size	Electrode distance	Electrode placement	Reference electrode	Orientation of the electrode	Parameters
Erector Spinae longissimus	10 mm	20 mm	2 finger widths laterals from the proc. spin. of L1.	Proc. spin. of C7.	Vertical	Mean power frequency (MPF), Median power frequency (MF), Root mean square (RMS) amplitude, Maximal Voluntary Contraction (MVC).
Erector Spinae iliocostalis	10 mm	20mm	1 finger width medial from the line of the posterior spina iliaca, superior to the lowest point of lower rib (level of L2).	Proc. spin. of C7.	Direction of the line between the posterior spina iliaca superior and the lowest point of the lower rib.	
Multifidus	10mm	20mm	Aligned from the caudal tip of posterior spina iliaca superior to the interspace between L1-L2 interspace at the level of L5 spinous process (2–3 cm from the midline).	Proc. spin. of C7.	In the direction of the line	

**Step 6- Field Evaluation (Post-Laboratory Validation):** Following laboratory validation and confirmation of mechanical safety, biomechanical performance, and user tolerance, the AgSuEx will undergo a structured field evaluation. Standardised agricultural bending tasks will be conducted in realistic or simulated field conditions. Participants will wear the device for 18–90 minutes [80] while performing traditional upright bending activities during harvesting. Observational data alongside usability and comfort factors will be collected to identify practical constraints in movement, fit, and task compatibility. Post-field FGDs will be conducted to assess user experiences and guide iterative device refinement before progressing to Phase II.

However, to assess the subjective experience, acceptability, and perceived productivity of farm labourers using the AgSuEx, a context-specific tool will be developed:a) A Work Productivity questionnaire, before and after device usage, will be designed to evaluate absenteeism, presenteeism, overall work productivity impairment, activity impairment, sustained standing and physical endurance, work speed, output, quality maintenance, economic impact, and outcome expectations. The questionnaire will be developed based on existing literature, pilot-tested with a small group of participants, and validated through expert review and iterative refinement to ensure content clarity, relevance, simplicity, and necessity.

#### Phase II: Pilot Testing and evaluation of the AgSuEx Integrated with Conventional Physiotherapy Vs Conventional Physiotherapy alone in Farm labourers with CLBP.

Phase II will enrol 24 farm labourers with CLBP (12 per group) assigned using convenience sampling to two parallel groups: 1) Intervention group- conventional physiotherapy combined with AgSuEx; 2) Comparator group- conventional physiotherapy alone, during the harvesting season in Wardha district. The six-week intervention will be followed by clinical and functional outcome assessments (Fig 4).

**Fig 4:**
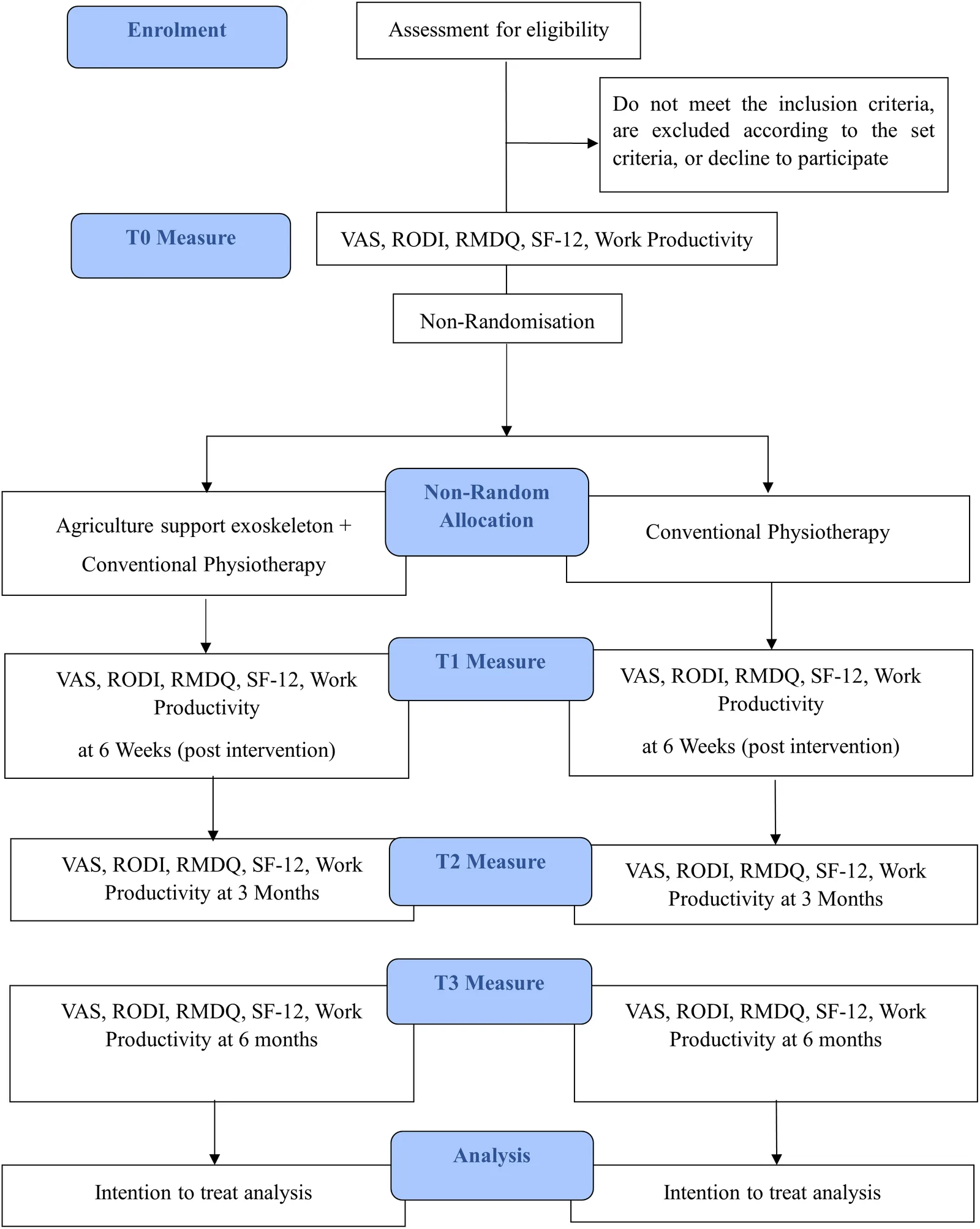
Participant Flow Diagram.

### Participant Flow Diagram for a quasi-experimental study

### Intervention Group: Conventional Physiotherapy with AgSuEx

The AgSuEx is hypothesised to reduce lumbar loading during harvesting tasks, while conventional physiotherapy addresses neuromuscular impairment, motor control deficits, and pain sensitisation. The combination is expected to provide synergistic benefit by simultaneously reducing biomechanical exposure and improving functional capacity.

Participants will receive: 1) integrated back-care education and ergonomic training delivered in Marathi through seminars, live posture demonstrations using models and posters, and group discussions at a central village location during the first two sessions. Content: postural training, load management, task rotation, basic spinal biomechanics, safe lifting techniques, and breathing strategies [81]; (2) Spinal mobilisation- posterior-to-anterior (PA) glides applied to hypomobile lumbar segments, one session per week for 5 weeks (10 minutes per session) [82]; (3) Core stabilisation exercises (CSE) targeting the transversus abdominis (TRA) and lumbar multifidus (LM) using a progressive four-stage protocol: Stage 1- isolated activation of TrA and LM by Abdominal Drawing-In Manoeuvre (ADIM) with Pressure Biofeedback; Stage 2- Side Bridge, Supine Extension Bridge, Prone Leg Raise; Stage 3- Alternate Arm & Leg Raise (Quadruped), Prone Bridge, Dead Bug; Stage 4- Bird Dog, Side Flank with Knee Flexion, Diagonal Curl-Up. CSE will be delivered 3 sessions/week for 6 weeks at 3 sets of 7 repetitions with a 7-second hold, 3-second inter-repetition rest, and 1-minute inter-exercise rest [83]; (4) Hip muscle stretching targeting hamstrings, iliopsoas, piriformis, and tensor fasciae latae (TFL): 3 times/week, 30-second stretch × 3 repetitions [83]; (5) AgSuEx use during harvesting- related bending tasks for 18–90 minutes per session, fitted individually based on standardised anthropometric measurements, while back-care education will not be provided during active exoskeleton use [80]. Supervised familiarisation sessions will precede independent use. Exercise dosage, intensity, progression, and session duration are elaborated in Table 6. A trained physiotherapist will supervise all interventions, and AgSuEx will be used only during designated agricultural activities and not during therapeutic exercise sessions.

**Table 6:** Conventional physiotherapy dosimetry (both groups: AgSuEx, additionally in the intervention group).

Exercise Components	Types	Dosimetry (FITT-VP)	AgSuEx used?
Spinal Mobilisation exercises [82]	Posterior to Anterior glides of the hypomobile lumbar segments	*Frequency*: one session per week (5 sessions total over 6 weeks) *Duration:* 10 minutes per session *Intensity:* Grade III-IV mobilisation, as tolerated by participant	No
Core Stabilisation Exercises (CSE) *Goal: Strengthen TrA and LM for lumbar stability* [79].	1^st^ stage: isolated activation of TrA & LM by Abdominal Drawing-In Maneuver (ADIM) with Pressure Biofeedback 2^nd^ stage: Side Bridge, Supine Extension Bridge, Prone Leg Raise 3^rd^ stage: Alternate Arm & Leg Raise (Quadruped), Prone Bridge, Dead Bug 4^th^ stage Bird Dog, Side Flank with Knee Flexion, Diagonal Curl-Up	*Frequency*: 3 times/week (18 sessions) for 6 weeks *Intensity*: 3 sets, 7 repetitions, 7-sec hold, 3-sec hold between repetitions and 1 min rest in between exercises (McGill 2015). *Time*: each session lasts for 30 minutes *Progression*: Increase reps to 10; Reduce hold phase and rest time; Advance positions: Supine → Sitting → Quadruped	No
Hip Muscle Stretching Exercises *Goal: Improve lumbar-pelvic mobility and correct muscle imbalances* [83].	Stretching of Hamstrings, Iliopsoas, Piriformis, and Tensor Fasciae Latae (TFL).	*Frequency*: 3 times/week for 6 weeks *Intensity*: 30-sec stretch, 10-sec rest, 3 repetitions (reps) per muscle *Time*: (15 min per session)	No
Integrated back care education and ergonomic training [81]	Theoretical and practical components: Postural alignment, load management principles, task rotation strategies, basic spinal biomechanics, ergonomic principles, safe lifting techniques, and breathing strategies to minimise lumbar muscle tension. Format: seminars, live demonstrations, and group discussions in Marathi.	*Sessions:* 1–2; delivered during the initial two intervention sessions *Weeks 1–3*: once weekly (45–60 minutes per session) *Weeks 4–6*: twice weekly (45–60 minutes per session)	No
AgSuEx use (intervention group only) [39]	Passive BSE is worn during harvesting-related farming activities (e.g., forward bending lifting, sustained stooping). Redistributes mechanical forces away from the lumbar spine to reduce muscular demand and paraspinal fatigue during agricultural tasks. *Fitting:* Individually adjusted per anthropometric measurements by trained research assistants under the supervision of a physiotherapist	*Duration*: 18–90 minutes per session during designated harvesting activities *Usage:* worn only during agricultural field activities; NOT used during therapeutic exercise sessions *Monitoring:* adherence tracked via usage logs, weekly field visits, and self-reports	Yes

### Comparator Group: Conventional Physiotherapy Alone

Participants will receive an identical conventional physiotherapy program to the intervention group, including the same exercise components, session frequency, duration, and back care education, but without the AgSuEx. This ensures that any differences between the two groups are due to the use of the AgSuEx rather than to variations in physiotherapy content or delivery.

### Outcome measures

#### Phase I primary and secondary outcomes.

Primary: Lumbar extensor muscle activation [surface electromyography (sEMG): MPF, MF, RMS amplitude, and MVC] during upright bending tasks with and without the AgSuEx.

Secondary: 1) trunk kinematics (IMU derived sagittal plane angular displacement during upright bending tasks; 2) interface pressure distribution (pressure mapping sensors to assess load transfer and localised pressure effects); 3) user-reported outcomes (SUS, QUEAD, and LPP scale) will be assessed at three key points: Phase 1 (lab testing), Phase 2 (pilot testing), and follow-up at 6 months.

#### Phase II primary and secondary outcomes.

Primary: 1) pain intensity (Quadruple VAS); 2) Work Productivity Questionnaire.

Secondary: 1) physical function [Roland Morris disability questionnaire (RMDQ)]; functional disability [Revised Oswestry Disability Index (RODI)]; 3) health-related QOL [Short Form-12 (SF-12)]; 4) Device-specific outcomes (SUS, QUEAD, LPP). All Phase II outcomes will be assessed at baseline, immediately post-intervention (6 weeks), and at follow-up time points of 3 and 6 months. Outcome assessors and data analysts will be blinded to group allocation.

### Feasibility outcomes

As this is a pilot study, feasibility outcomes are pre-specified as co-primary considerations alongside the clinical outcomes above, consistent with recommendations for pilot and feasibility trial reporting. The following progression criteria, summarised in **Table 7**, will be used to judge whether progression to a future, adequately powered, definitive randomised controlled trial is justified: (1) **Recruitment rate**: proportion of screened farm labourers who meet eligibility criteria and consent to participate, target ≥50% of those screened, recorded using a structured screening and recruitment log; (2) **Retention rate**: proportion of enrolled participants completing the 6-month follow-up assessment, target ≥80%, tracked through the participant flow diagram (Fig 4); (3) **Intervention adherence**: proportion of scheduled physiotherapy sessions attended and, for the intervention group, proportion of planned harvesting days during which the AgSuEx was worn for the prescribed 18–90 minutes per session (as specified in the Phase II intervention dosimetry, Table 6) during designated harvesting activities, target ≥80% for both, recorded using session attendance registers and AgSuEx usage logs; (4) **Usability and acceptability**: System Usability Scale (SUS) score ≥68 (the established threshold for acceptable usability) and Questionnaire for the Evaluation of Physical Assistive Devices (QUEAD) ratings in the favourable range, assessed in Phase I and re-assessed after Phase II field use; (5) **Safety and adverse events**: frequency, severity, and relatedness of device-associated and intervention-associated adverse events, with an a priori threshold that no serious adverse events related to the AgSuEx and no more than occasional, self-limiting, mild adverse events would be required to proceed to a definitive trial, captured through the adverse event monitoring system described under Data collection, management, and monitoring. These five feasibility parameters will be analysed descriptively (proportions, frequencies, and 95% confidence intervals) rather than through hypothesis testing, and will be reported using a traffic-light progression framework (green/yellow/red against each pre-specified target) to transparently guide the decision to proceed, proceed with modifications, or not proceed to a definitive randomised controlled trial.

**Table 7:** Feasibility Outcomes and Progression Criteria.

Feasibility domain	Operational definition	Target	Measurement method
Recruitment rate	Proportion of screened farm labourers who meet eligibility and consent to participate	≥50%	Structured screening and recruitment log
Retention rate	Proportion of enrolled participants completing the 6-month follow-up assessment	≥80%	Participant flow diagram (Fig 4)
Intervention adherence	Proportion of physiotherapy sessions attended; proportion of harvesting days with prescribed AgSuEx use	≥80% (both)	Attendance registers; AgSuEx usage logs
Usability and acceptability	SUS score and QUEAD ratings, assessed in Phase I and re-assessed after Phase II field use	SUS ≥ 68	SUS and QUEAD questionnaires
Safety and adverse events	Frequency, severity, and relatedness of device- and intervention-associated adverse events	No serious AEs	Adverse event monitoring system

Note: SUS = System Usability Scale; QUEAD = Questionnaire for the Evaluation of Physical Assistive Devices. Each domain will be reported using a traffic-light framework (green = target met, yellow = partially met, red = not met) to guide the decision to proceed to a definitive randomised controlled trial.

### Assignment of interventions and blinding

Phase I employs a within-participant, repeated-measures design, in which all participants perform tasks under both conditions (with and without the AgSuEx), serving as their own controls. No randomisation is applicable, and no allocation sequence is generated. Phase II uses convenience sampling and non-random allocation. Because farm labourers work in fixed geographical clusters (villages and farming communities) during the harvesting season, random allocation at the individual level risks contamination through social interaction and peer observation of device use. Therefore, allocation will proceed by geographical cluster: farm labourers recruited from one set of villages will be assigned to the AgSuEx-assisted conventional physiotherapy group (Intervention), and those from separate, non-overlapping villages within Wardha district will form the conventional physiotherapy-only group (Control). This approach minimises the risk of contamination between groups. Within each cluster, all eligible consenting participants will be allocated to the same group. The PI (GK) will assign participants to groups based on the village cluster to which they belong. The allocation plan will be documented in the Statistical Analysis Plan before any participant is enrolled. Sequential participant identification numbers will be assigned at enrolment within each group. A minimum of 3 and a maximum of 4 village clusters will be recruited per group (3–4 clusters in the intervention arm and 3–4 clusters in the control arm, depending on village level participant availability), with approximately 3–4 participants enrolled per cluster to reach the target of 12 participants per group. The exact number and composition of clusters per group will be recorder and reported in the participant flow diagram and baseline characteristics table. Because outcome measurements from participants within the same village cluster may be more similar to one another than to participants in other clusters (a design feature of cluster-based allocation), this within-cluster correlation will be explicitly accounted for in the primary statistical analysis using Generalized Estimating Equations (GEE) with robust standard errors clustered on village, with a liner mixed model (LMM) reported as a pre-specified sensitivity comparison (see Statistical Analysis for full justification and the non-convergence contingency plan, given the small number of clusters per group). This approach avoids treating clustered observations as independent and avoids anti-conservative (falsely narrow) confidence intervals that would otherwise result from ignoring the clustering. Baseline characteristics of both groups will be fully reported to evaluate comparability, and any important imbalances will be addressed through baseline-value adjustment within the linear mixed model described under Statistical Analysis. Baseline characteristics of both groups will be assessed and reported to evaluate comparability. This quasi-experimental approach was chosen because random allocation risks contamination among workers in proximity during harvesting, and is logistically impractical in this agricultural field setting [84]. Due to the visible nature of the AgSuEx, blinding participants and physiotherapists is not feasible. However, outcome assessors, who are independent of the intervention team, will be responsible for administering clinical questionnaires and recording outcome data, and data analysts conducting statistical analyses will be blinded to group assignment to reduce detection and analysis bias and to enhance the internal validity of the study. An independent researcher not involved in the study will enter collected data into separate coded datasheets. Participants will be instructed not to disclose their group allocation to outcome assessors. The same conventional physiotherapy content will be used for both groups, ensuring that the only difference between the groups is the use of AgSuEx in the intervention group.

### Participant adherence, fidelity, and concomitant care

Adherence to physiotherapy will be assessed through supervised sessions, organised exercise records, supervised exercise logs, and session attendance recorded by the treating physiotherapist. AgSuEx usage logs will record device duration and context, with specific descriptions of session duration and device utilisation context, and periodic monitoring will document correct fitting and donning/doffing of the device, along with session checklists. Standardised treatment guidelines will be used to ensure intervention fidelity, outlining physiotherapy procedures, exercise dosimetry, progression criteria, and exoskeleton use. All staff will undergo protocol-specific training before starting the study. At regular supervision meetings, adherence logs will be reviewed, discrepancies addressed, and adherence strengthened. Any deviation from the intended intervention will be recorded and reported. Those involved in both groups will be allowed to participate in routine daily life activities, agricultural work, and conventional medical care for CLBP (over-the-counter analgesics). New structured physiotherapy programmes, such as chiropractic manipulation, acupuncture, injection therapies, or other interventional studies targeting LBP that do not follow the study protocol, or initiation of other structured physiotherapy programmes outside the study protocol, will be restricted during the intervention period. Use of lumbar supports, braces, and any other type of wearable assistive device outside of the study-supplied AgSuEx will not be allowed. Concomitant care will be recorded using structured case record forms and included in the data analysis to address possible confounding effects.

### Data collection, management, and monitoring

All collected data will be coded with unique participant identification numbers to protect participant confidentiality and ensure anonymity. Quantitative data will be collected using electronic data collection tablets by trained research personnel and entered into password-protected spreadsheets by an independent data entry researcher not involved in intervention delivery or outcome assessment. Qualitative FGD data will be audiotaped, transcribed verbatim, and recorded as anonymised electronic files. All FGD participants will provide written informed consent that explicitly addresses: (i) audio-visual recording of the session; (ii) verbatim transcription of their statements; and (iii) potential use of anonymised, de-identified excerpts in research publications and conference presentations. Before any transcript excerpt is included in a publication, it will be rigorously de-identified to remove all potentially identifying information, including names, initials, nicknames, ages specified to within five years, village names, specific dates, household identifiers, and any other detail that could allow re-identification of an individual participant, in keeping with the data availability guidelines for human research participant data. Transcripts will not be published in full; only de-identified, anonymised excerpts will be reported. All data will be stored on secure institutional servers with limited access for authorised study personnel. Physical study documents will be kept in locked filing cabinets. Data will be stored for at least five years after publication, as stipulated by the institutional research governance.

A formal, independent Data Monitoring Committee (DMC) has not been constituted, given the exploratory, short-duration, non-pharmacological, minimal-risk nature of the study. Oversight will be undertaken by the Departmental Research Committee and DMC of RNPC under the IEC of DMIHER (DU). Actively monitored harms include musculoskeletal discomfort, exacerbation of LBP, skin irritation from exoskeleton contact, and balance or movement difficulties during the task, which will be assessed at each intervention session and at the outcome assessment visit through participant self-report and clinical observation by the supervising physiotherapist. Severity, duration, and perceived relationship to the intervention will be recorded. Participants will be instructed to report new or worsening symptoms, injuries, or health concerns to the research team immediately. All adverse events reported will be reviewed by the PI in consultation with the supervising physiotherapist and will then be recorded, coded by type and severity, and graded as mild events (requiring no intervention), moderate events (requiring temporary modification of the intervention), and severe events (necessitating discontinuation of the intervention or medical referral). Serious adverse events will be reported to the Ethics Committee. All harm data will be reported transparently in study publications and included in the final analysis.

### Participant Retention and Complete Follow-up

**Phase I-** Considering that this study is a single-visit experimental protocol, loss to follow-up is expected to be very low. Assessments will be conducted at appropriate times of the agricultural season to lessen the burden placed on participants and encourage participant retention. In case of participant discontinuation, until consent is withdrawn, all data collected up to the point of discontinuation will be preserved for analysis. Discontinuation reasons (e.g., discomfort, fatigue, personal reasons) will be recorded.

**Phase II-** Schedules for recruitment and intervention will be synchronised with participants’ agricultural work routines to minimise inconvenience and reduce dropout. Patients will be informed that they must complete all follow-up assessments, even if they stop or amend their participation in the intervention. Contact with participants will be maintained via telephone calls and physical reminders. Where participants cannot attend scheduled follow-up visits, outcome data will be collected at alternative times or locations where feasible. Reasons for non-adherence, withdrawal, or loss to follow-up will be documented. Outcome data for participants who discontinue will be retained and analysed according to the intention-to-treat principle.

### Stopping guidelines

Stopping guidelines are based on non-statistical safety considerations. The study may be paused or terminated if: i) Unexpected serious adverse events related to exoskeleton use occur; ii) Recurrent moderate adverse events indicate unacceptable discomfort or risk to participants; iii) Mechanical failure of the exoskeleton compromises participant safety. The decision to continue, modify, or terminate the study will be made by the PI in consultation with the DRC and the IEC of DMIHER (DU). All decisions and rationale will be documented and reported to the Ethics Committee.

### Criteria for modifying the intervention

The intervention will be modified in response to non-serious adverse events, participant-reported discomfort, or temporary symptom exacerbations. Modifications may include a reduction in exercise intensity or duration, adjustment of the exercise progression, alteration of exoskeleton use time, or a temporary suspension of device use while continuing conventional physiotherapy. All modifications will be made in consultation with the supervising physiotherapist and documented in the study records. Participants may request withdrawal from the intervention at any time without providing a reason, and such requests will be respected without prejudice to future care. Participants who discontinue the intervention will be encouraged to complete outcome assessments when feasible to include available data in the analysis.

### Statistical analysis

**Phase I:** Descriptive statistics (e.g., means, standard deviations, and ranges will be used to summarise participant parameters and all phase I outcomes. Data will be analysed using the Shapiro-Wilk test and visual inspection of Q-Q plots before inferential analysis, to assess the normality of continuous biomechanical data. For primary outcomes, within participant comparisons of lumbar extensor muscle activation (sEMG: RMS amplitude, MPF, MF) between exoskeleton-assisted and non-exoskeleton conditions will be performed using paired t-tests for normally distributed data or Wilcoxon signed-rank tests for non-parametric data. Secondary outcomes, including IMU-derived trunk kinematics, interface pressure distribution metrics, and validated questionnaire scores (SUS, QUEAD, LPP), will be analysed using the same paired analytical approach appropriate to data distribution. Effect sizes with 95% confidence intervals will be calculated for all primary biomechanical outcomes using Cohen’s d (paired). The level of statistical significance will be set at p < 0.05. Harms and adverse events will be summarised descriptively by frequency and severity. No formal multiplicity adjustment will be applied, given the exploratory nature of Phase I and the limited sample size, and no adjusted analyses are planned for this phase. All participants who complete at least one valid task condition with analysable biomechanical data will be included in the primary Phase I analyses. Participants will be analysed according to the condition performed (with or without AgSuEx). Participants who discontinue after partial data collection will be included in descriptive analyses using available data, unless they withdraw consent before study completion. Exploratory sensitivity analyses may be conducted to assess the robustness of findings by excluding incomplete or low-quality signal recordings; these analyses will be interpreted descriptively and not used for confirmatory inference. Qualitative FGD data from pre-field and post-field sessions will be analysed separately using inductive thematic analysis (Braun and Clarke 2006 framework) by two independent coders. A joint display integration matrix will map quantitative biomechanical findings against qualitative user-experience themes derived from FGDs, per MMR-RHS guidelines, operationalising the mixed-methods integration at the analysis stage.

**Phase II:** Data will be analysed using SPSS 27.0. Given the pilot, exploratory nature of Phase II, analyses will emphasise point estimates of treatment effect and their 95% confidence intervals rather than formal significance testing; p-values, where reported, are descriptive only. Continuous outcomes will be summarised as mean ± standard deviation at each time point. Because formal normality testing has very low power at this sample size, model choice will not be conditioned on a preliminary normality test. Because participants within the same village cluster may be more similar to one another than to participants in other clusters, this clustering must be accounted for; however, with only 3–4 clusters per group, a cluster-level random-effect variance component cannot be estimated reliably and risks convergence failure or a degenerate (near-zero) estimate. Given this, the pre-specified primary analytical approach for all continuous outcomes is Generalized Estimating Equations (GEE), fitted in SPSS (Analyze > Generalized Linear Models > Generalized Estimating Equations), with an independence working correlation structure, robust (sandwich) standard errors, and cases clustered on village. Each model will include fixed effects for group, time (a categorical factor with four levels: baseline, 6 weeks, 3 months, 6 months), and the group×time interaction, the primary parameter of interest, testing whether the pattern of change differs between groups, with the baseline outcome value included as a covariate. GEE was selected over a mixed model as the primary approach because it does not require estimating a cluster-level variance component, and its sandwich standard errors remain valid under working-correlation misspecification, an important property given the small number of clusters. As a pre-specified sensitivity analysis, the same fixed-effects structure will additionally be fitted as a linear mixed model in SPSS (MIXED procedure), with a random intercept for participant, restricted maximum likelihood (REML) estimation, degrees of freedom approximated using the Kenward-Roger method, and covariance structure selected by Akaike Information Criterion; a random intercept for village cluster will be added to this sensitivity model only if it converges without a singular or boundary fit. Estimates from the two approaches will be compared, and any material discordance discussed. If the primary GEE model fails to converge, the working correlation structure will first be simplified to exchangeable. If convergence failure persists, inference will instead be based on the mixed-model sensitivity analysis above, and this deviation will be transparently reported. ANCOVA will not be used, as it does not accommodate repeated measures. No covariates beyond the baseline value will be added unless strongly justified a priori and specified in the SAP before the data are examined. Between-group and within-group comparisons at each time point will be derived as estimated marginal means with 95% confidence intervals, rather than through multiple pairwise tests at each time point. Baseline comparability will be assessed descriptively, using Fisher’s exact test for categorical variables given the expected small cell counts. Effect size for all continuous outcomes will be reported as Cohen’s d with 95% confidence intervals, for both between-group and within-group comparisons. Given the sample size, these intervals are expected to be wide and should be interpreted as preliminary estimates to inform the design of a future, adequately powered trial, not as evidence of efficacy. Pre-specified minimally important clinical differences (1.5 cm for VAS; 3 points for RODI; 2.5 points for RMDQ; 5 points for the SF-12 physical component summary) will be used alongside these estimates to aid clinical interpretation. Full model estimates, observed means and standard deviations, and Cohen’s d for each outcome and time point will be reported in the results publication. The complete Phase II Statistical Analysis Plan, including the full GEE and sensitivity-model specifications above, will be finalised and locked before enrolment begins.

### Analysis population, intention to treat, and per protocol analyses

The primary analysis population will be defined according to the modified intention-to-treat (mITT) principle as is appropriate for quasi-experimental designs. All participants enrolled in and assigned to a study group who complete at least one post-baseline outcome assessment will be included in the analysis for the group to which they were allocated, irrespective of their level of adherence to the intervention protocol or any subsequent deviations. This approach is intended to provide conservative estimates of intervention effects reflecting real-world implementation conditions. The use of mITT rather than strict ITT is acknowledged as appropriate in this non-randomised design.

In addition to the mITT analysis, a per-protocol (PP) analysis will be conducted as a secondary exploratory analysis, including only those participants who complete the intervention as planned and adhere to predefined protocol criteria. The criteria for inclusion in the PP analysis will be specified a priori in the SAP. They will include minimum attendance at physiotherapy sessions (≥80%) and, where applicable, minimum documented exposure to the AgSuEx during harvesting season. Findings from PP analyses will be interpreted cautiously and reported as supplementary to the primary mITT analysis. Any discordance between mITT and PP results will be transparently reported and discussed.

### Handling of Missing Data

Missing outcome data are anticipated to be minimal given the short intervention duration and supervised nature of the study. The primary GEE analysis uses all available measurements from every participant at each observed time point, without requiring preliminary imputation; unweighted GEE is most robust to missingness that is unrelated to the outcome after conditioning on the fitted model (an assumption approximating missing completely at random, MCAR). Because the linear mixed model sensitivity analysis accommodates the weaker missing-at-random (MAR) assumption through likelihood-based estimation, comparison of the GEE and mixed-model estimates (see Statistical Analysis) also serves as an informal check on the plausibility of the MCAR assumption underlying the primary analysis; material discordance between the two would suggest MAR, rather than MCAR, missingness and would be discussed accordingly. The extent and pattern of missing data will be examined descriptively before analysis, and reasons for missingness documented for each participant with incomplete data. As a further sensitivity analysis, results from the primary GEE model will be compared with a complete-case analysis restricted to participants with data at all four time points. Multiple imputation will not be used, given its instability at this sample size. Any substantive discordance across these analyses will be reported. Participants will not be excluded from the analysis population solely for insufficient follow-up, and all inclusion and exclusion decisions will be disclosed transparently.

### Additional subgroup and sensitivity analyses

Since the pilot sample size will be 12 participants per group (n = 24 total), formal subgroup analyses for inferential purposes are not statistically appropriate and will not take place. Separating the sample by baseline characteristics would yield subgroups that are too small to allow meaningful between-group comparisons or valid statistical inferences. Therefore, no confirmatory subgroup analyses are planned. Nevertheless, further analyses will be performed. Exploration of descriptive patterns: Baseline attributes, including pain severity (Quadruple VAS score), duration of CLBP, sex, and intensity of occupational workload, will be analysed for each group, and the distribution patterns will be explored. Any observed heterogeneity will be noted and interpreted as a signal to inform stratification variables or covariate adjustment in future trials. These descriptions will not be treated as subgroup comparisons, and no p-values will be calculated or reported for these explorations. Sensitivity analyses for robustness: Sensitivity analyses will be conducted to assess the stability of primary findings to: (i) protocol deviations and per-protocol versus intention-to-treat populations; (ii) varying levels of adherence to physiotherapy sessions (defined as <80% versus ≥80% attendance); (iii) varying levels of AgSuEx exposure during harvesting tasks; and (iv) different missing data handling approaches (complete-case analysis versus the primary GEE-based analysis, with the linear mixed model sensitivity comparison, consistent with the approach specified under Handling of Missing Data). These analyses are methodologically appropriate regardless of sample size, as they assess the robustness of the analytical approach rather than the statistical power of group comparisons. Results will be presented alongside primary findings and clearly labelled as exploratory sensitivity checks. All findings from additional and sensitivity analyses will be transparently reported, clearly distinguished from primary analyses, and interpreted as descriptive and hypothesis-generating in preparation for future larger-scale trials.

### Statistical analysis plan and protocol

The present study protocol has been developed in accordance with the SPIRIT 2013 and SPIRIT 2025 expanded guidelines to ensure methodological transparency, reproducibility, and scientific rigour. A detailed Statistical Analysis Plan (SAP) will be finalised and locked before database closure and initiation of final analyses to minimise analytical bias and prevent data-driven decision making. The SAP will prespecify all primary, secondary, and exploratory analyses. Primary analyses will focus on between-group and within-group changes in pain intensity, work productivity, functional disability, physical function, and quality of life across predefined time points (baseline, post-intervention at 6 weeks, follow-up at 3 months, and long-term evaluation at 6 months). Secondary analyses will examine longitudinal trends using repeated-measures approaches to account for within-participant correlations over time. The effect size will be estimated, and confidence intervals and appropriate measures of variability will be calculated for all outcomes. Any substantive deviations from the projected statistical procedure will be explained, recorded, and transparently described in future publications. The protocol and SAP are designed in combination to maintain analytical integrity, minimise the risk of selective reporting, and improve the validity and interpretation of the study results. The full study protocol will be stored in the institutional repository of DMIHER (DU). Accumulation of appropriate materials (e.g., case record forms, data collection instruments) will be accessible on request to the corresponding author.

### Ancillary and post-trial care

During the study period, participants will continue to have access to routine healthcare services available within the community. Any medical needs arising outside the scope of the study interventions will be managed through standard clinical pathways. If participants experience discomfort, musculoskeletal exacerbation, or other minor issues related to study participation, the supervising physiotherapist will provide appropriate advice and referral. Following completion of the trial, participants will receive general guidance on back care and ergonomic principles, and will continue the beneficial exercises from the intervention. Participants who experience any harm directly attributable to study participation will be managed in accordance with institutional policies, and appropriate care will be provided through the host institution. No financial compensation or extended post-trial intervention will be provided; this will be clearly communicated during the consent process.

## Discussion

This protocol describes the first study, to the best of our knowledge, to integrate the co-design, engineering validation, and preliminary clinical testing of the AgSuEx within a structured physiotherapy framework for farm labourers with CLBP in a low-resource Indian setting. The protocol is positioned at the interface of occupational ergonomics, wearable assistive technology development, and rehabilitation science, a translational gap highlighted by several recent studies [7,40,41].

The agricultural biomechanical context is distinct from the industrial and military settings in which most exoskeleton research has been conducted. The extension of sustained upright-bending (trunk flexion >60°) during harvesting incurs lumbar compressive load estimated at three to eight times body weight, increasing cumulative musculoskeletal fatigue and risk of sustained injury [65]. Thamsuwan et al. (2020) demonstrated that passive BSEs could reduce lumbar muscle activity during farm-specific bending tasks in a controlled laboratory environment; however, this study lacked clinical integration and end-user validation [39]. The current protocol builds on this work by embedding the AgSuEx into a physiotherapy plan and evaluating it under real-world agricultural conditions. Koopman et al. (2020) evaluated a passive BSE using EMG-based biomechanical analysis and noted significant decreases in lumbar extensor activation during forward bending [78]. In line with their proposed approach, the current protocol uses sEMG (MPF, MF, RMS) and IMU trunk kinematics as biomechanical outcomes, thus providing mechanistic indicators for spinal load redistribution. However, unlike Koopman et al., an industrial example, the AgSuEx is specifically designed for Indian anthropometric dimensions and harvesting postures at an estimated cost of ₹3,000 compared to USD 100,000–130,000 used in commercial systems [40–42].

Selecting a passive, non-motorised exoskeleton architecture is also consistent with the growing consensus in this area of research. Ali et al. (2021) conducted a systematic review of BSEs and soft robotic suits. They concluded that passive devices are more practical in terms of cost, weight, and ease of application, which are important for their use in low- and middle-income farming contexts [46]. The active exoskeleton, although better engineered to support powered farming activity, needs an onboard power supply, sensor arrays, and maintenance infrastructure not designed for use in rural farming contexts, especially in developing countries. The AgSuEx’s modular Aluminium 6061-T6 frame (weight ≈1 kg), along with a multi-angle stay-lock hip joint and breathable harness system, will address this very limitation. Luger et al. (2023), in a full run-through trial conducted in a commercial BSE laboratory, identified comfort, user-friendliness, and compatibility with job tasks as the main reasons for adoption and long-term use [79]. The present protocol addresses these determinants prospectively through iterative co-design with farm labourers, validated usability instruments (SUS, QUEAD, LPP), and a structured field evaluation phase before clinical testing.

The participatory “by the farmers, for the farmers” design approach contrasts with the majority of published exoskeleton studies, which have excluded end users from the development process [7]. Arachchige et al. (2024), in a review of exoskeletons for agricultural WMSDs, specifically identified the absence of farmer-inclusive co-design as a key limitation restricting real-world applicability and adoption [7]. By embedding FGDs at both the pre-design and post-field evaluation stages, this protocol directly incorporates lived experiences, gender-specific postural demands, and cultural acceptability into device design and intervention protocols, conforming to the COREQ framework. The mixed-methods integration matrix, aligning biomechanical findings with qualitative user-experience themes, operationalises the MMR-RHS framework and will yield a richer understanding of feasibility barriers than quantitative data alone can provide.

Conventional physiotherapy combining spinal mobilisation, core stabilisation exercises, and hip muscle stretching has been shown in randomised controlled trials to reduce pain and disability in CLBP significantly [83,85]. Hrkac et al. (2022) demonstrated that supervised exercise therapy with a biopsychosocial approach produced significant and sustained improvements in VAS pain scores and Oswestry Disability Index in patients with non-specific CLBP [85]. Kim and Yim (2020) also found that core stability and hip activities enhanced physical function, and activity was significantly better in non-specific LBP [83]. These trials, however, were performed in clinical populations without prolonged occupational biomechanical exposure, suggesting the durability of the benefits over time. The AgSuEx has been proposed to overcome this limitation by redistributing lumbar load mechanically while performing harvesting duties throughout the intervention period, in an attempt to prevent a cycle of reinjury. This process can prevent physiotherapy-induced advantages from being preserved in occupationally active participants. While physiotherapy modulates neuromuscular dysfunction, motor control failure, and pain sensitisation, the AgSuEx alters biomechanical exposure directly, which causes the repetitive cycle of pain in occupationally active participants. Although this mechanism targets impairment and occupational exposure simultaneously, it is the first work to date to test that duality in agricultural populations, so this protocol is the main conceptual innovation. Back-care education that incorporates ergonomic training, present in each arm of the trial, has independent efficacy for reducing WMSDs among farm labourers [81] and will serve as an active comparator to verify that any additional effect noted in the treatment group is in fact attributable to the AgSuEx.

The quasi-experimental design reflects a pragmatic and well-justified methodological choice. In agricultural fieldwork settings, random assignment of workers within the same proximity during harvesting is prone to contamination, peer effects, and co-intervention bias. Quasi-experimental designs with multiple assessment time points, including statistical adjustment for baseline differences, are generally accepted in occupational and public health intervention research when such constraints are available [84]. Repeated outcome measures at post-intervention and follow-up time points would further enhance the protocol by allowing examination of both short-term effects and maintenance of gains, a clinically important question for occupational populations with persistent exposures.

Several sources of bias arising from the non-randomised design require explicit acknowledgement and mitigation. First, **selection bias** is the primary threat in this design: convenience sampling and cluster-based non-random allocation mean that systematic differences between villages in farming intensity, access to healthcare, pain chronicity, or health literacy could produce non-comparable groups at baseline, confounding any between-group comparison. Mitigation strategies include: comprehensive baseline characterisation of both groups across all demographic and clinical variables; publication of full baseline comparison data regardless of statistical significance; and baseline-value adjustment for the outcome measure within the linear mixed model used for the primary analysis. Second, **performance bias** is unavoidable because the AgSuEx is visible: participants in the intervention group will be aware of receiving an additional device, and treating physiotherapists cannot be blinded to group assignment. This may introduce differential motivation, placebo, and Hawthorne effects. Mitigation: identical physiotherapy content is delivered to both groups; the comparator group receives an equivalent supervised programme to maintain engagement and minimise differential attention. Third, **detection bias** is reduced through independent, blinded outcome assessment: outcome assessors who administer clinical questionnaires and analysts who conduct statistical analyses will both be blinded to group assignment and will not have access to group allocation codes until analysis is complete. Participants are instructed not to disclose their group to the assessor. Fourth, **attrition bias** is addressed through the modified ITT analysis, which includes all enrolled participants with at least one post-baseline assessment regardless of adherence, and through active retention strategies including phone follow-up and flexible scheduling of outcome assessments. Differential dropout between groups will be reported and examined as a sensitivity analysis. Fifth, **contamination bias**, where control-group participants observe or imitate intervention-group behaviour, is minimised through geographical cluster allocation, where intervention and control villages are spatially separated.

The small sample size (n = 12 per group), while consistent with pilot study guidance [61,62], will limit statistical power and preclude confirmatory inference. This is acknowledged as inherent in early-phase pilot research and does not diminish the study’s primary purpose of generating feasibility estimates and preliminary effect data. Biomechanical evaluation under a standardised laboratory task cannot fully replicate the variability of extended agricultural workdays. Seasonal and weather variations may influence adherence and device use during harvesting. All these limitations will be transparently reported in accordance with the TREND checklist and will be used to inform the design of a future definitive randomised controlled trial in which allocation concealment, adequate power, and minimisation of the biases identified above will be fully addressed.

Although these limitations are significant, this research is anticipated to provide essential preliminary data for a future definitive RCT. The combination of engineering validation with biomechanical, clinical, and usability outcomes provides a clear framework for the iterative development of the AgSuEx. The results of this protocol will contribute to optimising design parameters, predicting sample sizes for future trials, and informing the design of time-tested field studies on the long-term clinical effectiveness, work productivity, adoption, and cost-effectiveness of AgSuEx. Thus, the protocol operationalises the Medical Research Council (MRC) framework for the design and validation of new interventions, with pilot research as an integral phase of evaluation before definitive efficacy testing.

## Conclusion

This protocol describes an interdisciplinary, phase-wise study aimed at the development, validation, and initial evaluation of an affordable, farmer-oriented AgSuEx, complemented by conventional physiotherapy, for farm workers with CLBP in rural Maharashtra, India. Utilising physiotherapy-based rehabilitation and task-tailored mechanical lumbar support with participatory co-design, the investigation will present preliminary evidence of whether a combined ergonomics and rehabilitation approach is associated with clinically significant improvements in pain, disability, work productivity, and quality of life in this under-resourced occupational population. The implications will help expand the evidence-based therapeutic and ergonomic interventions for agricultural assistive technologies and contribute towards future larger trials.

## Supporting information

S1 Appendix MMR-RHS checklist. (PDF)

S2 Appendix Coreq-checklist. (PDF)

S3 Appendix Trend-checklist. (PDF)

S4 Appendix SPIRIT-checklist. (PDF)

S5 Appendix TIDieR-checklist. (PDF)

S6 Appendix Structured focus group interview guide. (PDF)

## Abbreviations

VAS: visual analogue scale
RMDQ: Roland Morris Disability Questionnaire
RODI: Revised Oswestry Disability Index
SF-12: Short Form-12
SUS: System Usability Scale
QUEAD: Questionnaire for the Evaluation of Physical Assistive Devices
LPP: Local Perceived Pressure
PT: physiotherapy
FGD: focus group discussion
