# Supplementary material for: Development, validation, and testing of an agriculture support exoskeleton in adjunct to conventional physiotherapy for farm labourers with chronic low back pain: A two-phase, mixed-methods, quasi-experimental pilot study protocol
Source: PLoS One. 2026 Nov 6;21(11):e0361352. doi: 10.1371/journal.pone.0361352 (PMC13742883; doi:10.1371/journal.pone.0361352)
Supplement: Supplementary file 6 [file pone.0361352.s006.pdf]

Focus group discussion guide

Overview of focus group discussion procedures

Two focus group discussions (FGDs) will be conducted as part of Phase I of this study: (1) a Pre-Field Testing FGD conducted before participants use the Agricultural Support Exoskeleton (AgSuEx), and (2) a Post-Field Testing FGD conducted immediately after 6 weeks of treatment. Each FGD will comprise two sex-stratified groups (one male, one female) of 8-9 participants each, facilitated by a trained moderator. A co-facilitator will take field notes and operate the audio/video recorder throughout each session. Each session will last approximately 60 minutes and will be conducted in the participants' local language. All sessions will be audio-recorded following written informed consent from each participant.

The moderator will facilitate open, non-directional discussion. Participants should feel free to agree or disagree with one another. There are no right or wrong answers. All responses will be treated with strict confidentiality. Participants will be addressed by pseudonyms or participant codes, and no identifying information will be disclosed in any publication or report.

Ground rules for all sessions (read aloud by moderator)

Before we begin, I would like to share a few guidelines that will help our discussion run smoothly:

- Please speak one at a time so that everyone's contribution can be recorded.
- There are no right or wrong answers, we are interested in your personal experiences and opinions.
- Please feel free to agree or disagree with what others say.
- You may choose not to answer any question you are not comfortable with, and you may leave at any time without consequence.
- Everything shared in this session will remain strictly confidential and will only be used for research purposes.

Part 1: Pre-field testing - focus group discussion guide

Low Back Pain and Agricultural Exoskeleton Implementation among Farm Workers

Introduction to participants (read aloud by moderator)

Welcome, and thank you for participating in this focus group session. Namaskar. My name is ______________, and I am a researcher from the Department of Musculoskeletal Physiotherapy, Ravi Nair Physiotherapy College, Datta Meghe Institute of Higher Education and Research (DMIHER), Wardha. Thank you very much for joining us today. Today's discussion is part of a research study that we are conducting in your community. The purpose of this study is to understand the physical challenges that you face during your daily farming work, particularly those related to back pain and body strain, and to learn what kinds of support

or tools might be helpful for you. We are especially interested in your personal experiences and honest opinions. We are not evaluating or testing you in any way. Everything you share today will be kept strictly confidential. Your name will not be recorded or used in any research publication. You will be referred to only by a participant code number. Participation is entirely voluntary. We will be recording this session with your permission, so that we can carefully review your responses later. Only the research team will have access to these recordings. The session will last approximately 60 minutes.

Please ask me at any time if you have a question, or if you would like me to clarify anything. You may also choose not to answer any question, or to leave the session at any time, without any effect on your participation in the broader study.

Do you have any questions before we begin?

Engagement

Q1. Farming background and experience

Please introduce yourself by sharing how many years you have been farming and the main types of farming activities you are involved in.

Moderator probes: Type of work performed most frequently (e.g., sowing, weeding, harvesting, lifting, carrying). Seasonal or year-round work. Whether you work on your own land or as a hired labourer.

Q2. Daily work routine and workload

Can you describe a typical working day on your farm, including the main tasks you do and approximately how much time you spend on each?

Moderator probes: Total working hours per day. Tasks that take the longest time. How work changes across seasons, particularly during harvesting.

Exploration

Q3. Physical demands and task distribution in the community

Which farming tasks do you find most physically demanding? Do you notice any differences in how physically demanding tasks are shared among the farming community where you work?

Moderator probes: Tasks that involve prolonged bending, lifting, or carrying heavy loads. Differences in physical strain depending on the nature of the work. Whether some tasks feel harder on the body than others.

Q4. Posture and work-related physical strain

How do your usual working postures, such as prolonged bending or squatting while harvesting, affect your physical comfort and health overtime?

Moderator probes: How long you typically maintain bent or stooped postures during a working day. Which body regions feel most strained or fatigued (e.g., lower back, neck, shoulders, knees). Whether the strain worsens as the workday or season progresses.

Q5. Low Back pain and its impact on your daily life

Have you experienced pain or discomfort in your lower back related to farming work? If yes, which activities or times of year make it worse, and how does it affect your ability to work or carry out daily activities?

Moderator probes: When the pain first started and how long it has lasted. Which specific tasks, such as bending for harvesting or lifting loads, worsen the pain. Whether pain has caused you to slow down, take rest breaks, or stop work entirely. How it affects activities at home, sleep, or personal care.

Q6. Coping strategies, healthcare access, and decision-making

What steps do you currently take to manage or reduce your low back pain? What difficulties, if any, do you face in getting the right treatment or information?

Moderator probes: Home remedies, medications, back supports, physiotherapy visits, or changes in work habits. Barriers to seeking treatment such as cost, distance from a health facility, lack of time, or limited awareness of available options. Who in your household or community typically makes decisions about when to seek medical treatment or when to rest from work.

Q7. Needs and suggested improvements in farming work

What changes in farming practices, tools, or working methods do you feel are most needed to reduce physical strain on the body and prevent back problems among farm workers in your community?

Moderator probes: Types of support or modifications that workers feel would help most. Any changes that you or others in the community have already tried. What would help you continue farming with less pain or physical strain.

Moderator instruction

"*Transition to the next section by saying: 'We have been discussing the physical challenges of farming work and back pain. I would now like to ask you about a possible solution, a wearable back support device that is being developed specifically for farmers like yourselves. You do not need to have any previous knowledge of such devices; we are simply interested in your thoughts and questions.*"

Exit

Q8. Awareness, acceptance, and expectations for a wearable back support device

Have you ever heard about or used a wearable device designed to support your back during physically demanding work? What features or conditions would make such a device comfortable, safe, and acceptable for use during daily farming activities?

Moderator probes: Any prior awareness of back belts, lumbar supports, physiotherapy aids, or wearable assistive technologies. Concerns about weight, heat or sweating, comfort, cost, ease of putting on and taking off, and safety. What an ideal support device would look and feel like. Whether you would feel comfortable using such a device in front of other community members or fellow workers.

Session Closing, Pre-Field FGD (Read Aloud by Moderator)

Thank you very much for your time and for sharing your experiences so openly. Your insights are extremely valuable and will directly shape the design of the back support device and the physiotherapy programme that will be tested in this study. We will now close the recording. If you have any further questions about the study, please do not hesitate to contact the research team. We appreciate your participation.

Part 2: Post-field testing - focus group discussion guide

Evaluation of Agricultural Support Exoskeleton (AgSuEx) after Field Use

Introduction to participants (read aloud by moderator)

Namaskar. Welcome back, and thank you for returning to take part in this second focus group discussion. As you know, you have recently used the AgSuEx during your farming work. Today, we would like to hear about your actual experience of using the AgSuEx: what worked well, what did not, how it felt during different types of work, and what changes or improvements you feel are needed. As before, there are no right or wrong answers. We are not evaluating your performance, we are evaluating the device, and your honest feedback is essential to improving it. Everything you share will remain strictly confidential and will be used only for research purposes. You may choose not to answer any question, or to leave at any time. With your permission, we will again record this session for research purposes only. The session will last approximately 60 minutes.

Do you have any questions before we begin?

Engagement

Q1. Overall experience with AgSuEx

Can you describe your overall experience using the AgSuEx during your farming work?

Moderator probes: How long and how often you wore the device. Which farming tasks you used it for most frequently. Your first impressions when you put it on, and how your experience changed over time.

Exploration

Q2. Satisfaction and perceived usefulness

How satisfied are you with the back support provided by the AgSuEx, and to what extent did it meet your needs during farming work?

Moderator probes: Specific situations or tasks where you found it most helpful. Any situations where the support felt insufficient or unnecessary. Your perceptions of the device's weight, bulkiness, build quality, and durability.

Q3. Compatibility with farming activities

How well did the AgSuEx fit into your regular farming tasks? Did it affect your speed, flexibility, or ability to complete your work?

Moderator probes: Tasks where the device worked well and tasks where it interfered with your work. Specific difficulties with bending, lifting, walking, or maintaining prolonged postures while wearing it. Whether you needed to adapt your working style to use it.

Q4. Physical comfort, safety, and freedom of movement

How comfortable and safe did you feel wearing the AgSuEx during extended periods of work?

Moderator probes: Pressure points, rubbing, or skin irritation from straps or the frame. Heat, sweating, or discomfort related to wearing the device in the field. Feelings of imbalance, restriction of movement, or any new pain or discomfort caused by the device. Whether you ever stopped using the device during work because of discomfort or safety concerns.

Q5. Fatigue and physical workload

Compared to working without the AgSuEx, how did wearing the AgSuEx affect your fatigue levels and physical strain on your body during the working day?

Moderator probes: Changes in how your back, shoulders, or legs felt after a full day of work. Whether any tasks required noticeably more or less physical effort while wearing it. At what point during the working day, early, mid, or late, you noticed any differences in strain or fatigue.

Q6. Work performance and productivity

Did you notice any changes in your pace, endurance, or ability to complete your farming tasks while using the AgSuEx?

Moderator probes: Whether you were able to work longer without taking rest breaks. Any delays, interruptions, or difficulties completing tasks while wearing the device. Specific tasks where you noticed a clear benefit or limitation in performance.

Q7. Ease of use, design and social acceptability

How easy was it to put on, remove, and adjust the AgSuEx? What are your thoughts about its design and about using it in a community or social farming environment?

Moderator probes: Whether you needed assistance to fit or adjust the device. Difficulty or comfort with the straps, harness, or frame components. Your views on the device's appearance and whether it felt appropriate for use in the field. How other farmers, family members, or community members reacted when they saw you wearing it. Whether you felt comfortable, self-conscious, or proud wearing it in front of others.

Moderator instruction

"*Transition to the Exit question by saying: 'We have heard a great deal about your experiences using the AgSuEx. I would like to finish by asking you to look ahead, to think about the future of this device and what would help it become a practical, everyday tool for farmers*."

Exit

Q8. Future use and recommendation for improvement

Would you be willing to continue using the AgSuEx in the future? What changes or improvements would make it more practical, comfortable, and acceptable for regular daily use in farming?

Moderator probes: The main barriers to continued use, such as cost, discomfort, weight, need for maintenance, or social concerns. Reasons why you might stop using it, or conditions under which you would be most likely to continue. Any concerns about becoming dependent on the device, or conversely, feelings of confidence or empowerment from using it. Any additional suggestions, concerns, or ideas that have not yet been discussed.

Session Closing, Pre-Field FGD (Read Aloud by Moderator)

Thank you sincerely for your time, your patience throughout this study, and for the honesty and detail with which you have shared your experiences today. Your feedback will directly contribute to improving the design and implementation of this device for future users. We will now close the recording. Please do not hesitate to contact the research team if you have any further questions or concerns. We are grateful for your participation.
